# Cohort Trends in the Association Between Sibship Size and Educational Attainment in 26 Low-Fertility Countries

**DOI:** 10.1007/s13524-020-00885-5

**Published:** 2020-06-22

**Authors:** Seongsoo Choi, Riley Taiji, Manting Chen, Christiaan Monden

**Affiliations:** 1 Department of Sociology, Yonsei University, Seoul, South Korea; 2 Department of Sociology, University of Oxford, Oxford, UK; 3 Nuffield College, Oxford, UK; 4 Leverhulme Centre for Demographic Science, University of Oxford, Oxford, UK

**Keywords:** Siblings, Sibship size, Educational attainment, Cross-national comparisons

## Abstract

Children with many siblings have lower average educational attainment compared with children raised in smaller families, and this disadvantage by sibship size has been observed across many countries. We still know remarkably little, however, about how sibship size disadvantage has changed within countries and how such trends vary across countries. Using comparative data from 111 surveys from 26 low-fertility countries, we find an overall trend of growing sibship size disadvantage across cohorts in the majority of countries: between the 1931–1940 birth cohort and the 1971–1980 birth cohort, 16 of 26 countries showed a statistically significant increase in sibship size disadvantage in education, while only two countries showed a significant reduction in sibship size disadvantage. The disadvantage in years of education associated with having an additional sibling increased remarkably in post-socialist (0.3) and East Asian countries (0.34) and, to a lesser extent, Western European countries (0.2). In contrast, this disadvantage showed little change in Nordic countries (0.05) and even decreased in Anglo-Saxon countries (–0.11). We discuss explanations and implications of our comparative evidence in the context of the intergenerational transmission of education.

**Electronic supplementary material:**

The online version of this article (10.1007/s13524-020-00885-5) contains supplementary material, which is available to authorized users.

## Introduction

The negative relationship between family size and children’s educational outcomes in developed countries is one of the most frequently confirmed empirical patterns in social stratification and demographic research (Steelman et al. 2002). As early as the 1960s, Blau and Duncan (1967) observed that men from smaller families tended to have higher educational attainment than men from larger families, remarking that a man’s career chances are highly impacted by the size of his parental family. Since then, negative associations between the number of siblings (hereafter, sibsize) and various educational outcomes have been reported for several developed countries, with mechanisms such as resource dilution (Blake 1989; Downey 1995, 2001) and differential fertility (Dalla-Zuanna 2007; Kasarda and Billy 1985) commonly being cited.

However, the shape and strength of the negative relationship between sibsize and educational attainment—or whether there is a negative relationship at all—will likely depend in part on the context in which the relationship is studied. The general trends toward both smaller family sizes and increased educational attainment occurring throughout most industrialized societies over the past century portend temporal variations in the shape and strength of this association. Yet, our knowledge about these trends and their cross-national variation remains incomplete: current evidence has relied on a few country cases, such as the United States (Blake 1989; Gibbs et al. 2016). For example, analyzing data from General Social Surveys from 1972 and 2010, Gibbs et al. (2016) found that the educational disadvantage associated with sibsize in the United States nearly halved for individuals born in the 1900s compared with those born in the 1960s. An important question, then, is whether the U.S. experience applies to other societies, too.

Evidence from other countries suggests that the decline in the sibsize penalty observed in the United States may not be easily generalizable to other contexts. For example, Marteleto and De Souza (2012) showed that in Brazil, the association between sibsize and education became increasingly negative as fertility fell and education expanded during the second half of the past century. In China, Lu and Treiman (2008) reported that the sibsize penalty in educational attainment was smaller in periods dominated by equalization policies and larger when policies enhancing market competition and inequality were dominant. This suggests an increasingly strong negative association between sibsize and educational attainment as the country embraced a more market-based economy. Maralani (2008) documented that in the urban areas of Indonesia, the association between family size and educational attainment was positive for older cohorts but became more negative for recent cohorts, suggesting that the context of socioeconomic development in a society moderates the sibsize penalty in educational attainment. Maralani also found no observable sibsize penalty in rural areas of Indonesia, providing further evidence that the negative sibsize-education association may not necessarily hold in less-developed contexts. Similar results were also found for Malaysia (Pong 1997) and Taiwan (Parish and Willis 1993).

This mix of results suggests that the pace and nature of changes in the association between sibsize and education have been far from uniform across different social contexts and that we cannot draw conclusions from a limited sample of countries—or, at least, we do not know whether we can. In a similar spirit, Lu and Treiman (2008:813) maintained that “the negative effect from the number of siblings is neither universal nor inevitable” and that it is “contingent on demographic, socioeconomic, and political factors external to the family that influence both the availability of resources and their internal allocation within a family.” In other words, the relationship between sibsize and educational outcomes is a topic that should be examined through a comparative lens. Nonetheless, little systematic comparative research has explored this issue, presumably because of a lack of readily available data.

We aim to fill this void by analyzing a new comparative data set comprising 111 surveys from 26 low-fertility countries. Our data set covers 536,124 adult individuals from 167 country-cohort samples spanning the course of the twentieth century. In our analyses, we first show how average sibsize has changed from those born in the early twentieth century to those born in 1980s. Following this, we examine how the disadvantage in educational attainment associated with an additional sibling has changed across birth cohorts in our 26 countries. Our analyses reveal that the negative association between sibsize and educational attainment strengthened in the majority of countries. However, we also observe substantial variation in these trends, both between and within regions. The trend toward a stronger sibsize penalty is most prominent in post-communist and East Asian countries, whereas stable or even decreasing trends are seen in Anglo-Saxon countries. We also find growing sibsize disadvantages in countries where the gap in parental education between smaller and larger families has increased, suggesting that growing differential fertility is likely a key factor driving increasing sibsize disadvantage.

## Relationship Between Sibship Size and Educational Attainment

Researchers have applied two theoretical perspectives to explain why family size matters for the outcomes of children. One perspective emphasizes the importance of economic, cultural, and interpersonal resources in the family that are shared among siblings and benefit a child’s development. Family resources dilute as the number of siblings competing for these resources increases. To the extent that a child’s outcomes are influenced by the input of these resources, resource dilution (RD) predicts a negative impact of having an additional sibling on educational attainment (Downey 1995, 2001; Steelman et al. 2002).

The other perspective highlights the role of differential family size by parents’ socioeconomic status (SES). If families that choose to have more children tend to have a lower SES than those who opt for lower parities, the children in those large households might end up with lower educational attainment independent of the effect of sibsize per se (Dalla-Zuanna 2007; Kasarda and Billy 1985). Thus, part of the observed negative association between sibsize and child outcomes is capturing the effects of parental characteristics that affect both parents’ fertility preferences and children’s socioeconomic outcomes (Black et al. 2005), such as parents’ cognitive skills (Rodgers et al. 2000) and career aspirations (Upchurch et al. 2002), both of which are correlated with parents’ SES.

These perspectives represent two sides of the debate on the causal effect of sibsize. Explanations emphasizing how family resources are pooled and allocated suggest a causal effect of family size, whereas differential fertility posits that selection or confounding generates the negative sibsize-education association. Several studies that tried to identify a causal effect of sibsize using twin births or gender composition of the firstborn children found that a considerable portion of the observed association between sibsize and educational attainment is attributable to unobserved factors (Black et al. 2005; Conley and Glauber 2006; Guo and VanWey 1999; Marteleto and De Souza 2012). However, there is also evidence suggesting that the causal effect of family size on education is not uniform across different social and economic contexts (for education, see Marteleto and De Souza 2012; for other child outcomes, see Öberg 2017). More recently, the validity of twins as an instrument in this literature has been seriously criticized (Bhalotra and Clarke 2016).

## Variations Over Time and Societies: Explanations and Hypotheses

How, then, does the relationship between sibsize and education change over time, and how does this vary across societies? Previous studies have suggested a few candidate factors. Building on these factors, we develop four hypotheses that predict cross-national variation in trends of the sibsize penalty in educational attainment.

First, previous research has highlighted the importance of public policies as a moderator of the role of family resources (Steelman et al. 2002). Public policies that support education and families are likely to compensate for disadvantages of children from larger families. For example, Gibbs et al. (2016) showed that the negative effect of sibsize on educational attainment in the United States was largest in states where per capita spending on higher education was lowest, suggesting that state resources and policies can offset the impact of RD. They framed this as *conditional RD* and suggested that “the expanding size of state-sponsored investments in education and related programs that unlink family background from educational opportunities” explain “the decline in the relationship between sibship size and educational attainment over time” (Gibbs et al. 2016:738). Cross-sectional evidence on between-country variations in sibsize and student performance using Programme for International Student Assessment data also lends support to the importance of public policies in compensating educational disadvantages associated with a large number of siblings, revealing a trend of weaker sibsize penalties among countries (e.g., Nordic) with more inclusive public policies (Park 2008; Xu 2008). This perspective, therefore, predicts that the sibsize penalty will be weaker in countries where public policies are more inclusive—such as Nordic and, to a lesser extent, Western European countries—and stronger in countries where public policies are less inclusive.

This same logic can also be applied to within-country variation. The development of the welfare state and mass public education over the past century is likely to have weakened the disadvantage associated with large family size. By extension, a strengthening trend in disadvantage will have likely occurred in countries that experienced an opposite direction of change, such as post-communist countries, which underwent the transition from a state-communist regime to a market-based economy (e.g., China; Lu and Treiman 2008). If RD and the consequent sibsize penalty is moderated by the inclusiveness of public policies and related processes of decommodification, we can put forward the following two hypotheses:

- *Hypothesis 1 *(H1): Countries experiencing the expansion of social and welfare policies will show an increasingly small educational disadvantage associated with having an additional sibling across birth cohorts.
- *Hypothesis 2 *(H2): Countries that experienced a transition from a state-socialist to a market-based economy will show an increasingly large educational disadvantage associated with having an additional sibling across birth cohorts.

Another explanation highlights structural changes in schooling systems and labor markets. Many researchers have used economies of scale to explain why a larger sibsize is not necessarily harmful for children’s education (Deaton and Paxson 1998; Qian 2009). However, the effectiveness of economies of scale in buffering the sibsize penalty depends on the educational systems and labor markets in which parents and children are embedded. For example, Maralani (2008) maintained that in a less developed context where children are usually expected to contribute to the family economy, siblings can be resources that share domestic and market labor and ultimately help overcome barriers to education. Such a mechanism may not be present in more developed societies. Parish and Willis (1993) also argued that as a society develops, children from large families tend to have more difficulties balancing education and work due to rising opportunity costs, which translates to an increasingly large sibsize disadvantage in education. This explanation is closely related to institutional incentives for quantity-quality trade-offs (Angrist et al. 2010; Becker and Lewis 1973), which predict that as success in the labor market becomes more tightly coupled with success in school, benefits from a large pool of siblings wane, and those from a smaller pool grow. Taken together, this second explanation suggests that we can expect more notable increases in sibsize disadvantage in education in societies that experienced rapid transitions of this kind—for example, late-industrializing countries in East Asia and Southern Europe.

- *Hypothesis 3* (H3): Countries that experienced rapid social and institutional transformation of markets and education are likely to show an increase in the educational disadvantage associated with having an additional sibling across birth cohorts.

The third explanation suggests that widening differential fertility by parental SES can increase the sibsize disadvantage in children’s educational outcomes (Van Bavel 2006). As discussed earlier, more marked fertility limitation by high-SES relative to low-SES families (Dalla-Zuanna 2007) can be an important driver of the sibsize gap in education. We can thus expect a growing sibsize penalty in education in contexts where gaps in family size by parent’s SES have widened.

- *Hypothesis 4* (H4): Countries that experience a widening family size gap across parents’ SES groups will show an increase in educational disadvantage associated with having an additional sibling across birth cohorts.

In Table 1, we map these four hypotheses to six cross-national, low-fertility regions. We expect that Nordic countries will show relatively small differences in educational attainment across sibsize groups across cohorts because of the buffering role of inclusive public policies (H1). These inclusive policies may also mean that differential fertility plays a less important role in these countries. In contrast, we predict notable increases in sibsize disadvantages among post-communist countries because of their transition to market-based economies (H2). Similarly, we expect rising sibsize disadvantage among East Asian and, to a lesser extent, Southern European countries (H3). With regard to change in the SES gradient in sibsize (H4), there is insufficient comparative evidence to support specific regional predictions. However, we can test this by examining to what extent changes in the gap in parental status between large families and small families across sampled countries explain variation in the cohort trends in sibsize disadvantage.

**Table 1:** Mapping four hypotheses about trends in changing sibship size disadvantage in education across regions

	Public Policies			
	Inclusive Social and Welfare Policies	Transition From Socialist to Market Economy	Rapid Restructuring in Markets and Education	Growing SES Gap in Family Size
Western Europe	–			
Northern Europe	–			
Southern Europe			+	
Central-Eastern and Eastern Europe		+		
Former USSR		+		
Anglo-Saxon				
East Asia		+ (China)	+	

*Note*: – indicates weakening sibsize disadvantage; + indicates strengthening sibsize disadvantage.

Last, we highlight the possibility that changing distributions of sibsize and educational attainment can also bring a change in sibsize disadvantage in education. The estimated coefficient of sibsize (call this, *x*) in the regression of educational attainment (call this, *y*) is the product of the standardized correlation between *x* and *y* and the ratio of the standard deviations (SD) of *y* and *x*:

1 $$ \hat{\upbeta}=\frac{\mathrm{Cov}\left(x,y\right)}{\mathrm{Var}(x)}=\mathrm{Corr}\left(x,y\right)\frac{\mathrm{SD}(y)}{\mathrm{SD}(x)}. $$

The three explanations discussed earlier reflect theoretical mechanisms explicating a change in Corr(*x*, *y*) on the right side, being ignorant of how a change in $\frac{\mathrm{SD}(y)}{\mathrm{SD}(x)}$ will affect $\hat{\upbeta}$. Equation (1), however, indicates that a change in the variance of *x* contributes to change in the coefficient $\hat{\upbeta}$, which has been used to gauge the educational disadvantage associated with larger sibsize (Downey 1995; Gibbs et al. 2016; Lu and Treiman 2008). Similarly, a change in the variance of *y* between two time points also contributes to a change in $\hat{\upbeta}$. The general trends toward both subreplacement fertility levels as well as increasing educational attainment among industrialized countries therefore suggest shrinking variances of *x* and *y*.^1^ However, countries will likely vary widely in the scale and pace of these changes in fertility and education. In other words, how $\frac{\mathrm{SD}(y)}{\mathrm{SD}(x)}$ changes between two time points (or two birth cohorts) is also likely to vary across countries. This reasoning then suggests that separating the two components of $\hat{\upbeta}$, Corr(*x*, *y*) and $\frac{\mathrm{SD}(y)}{\mathrm{SD}(x)}$, will help to better understand trends in sibsize disadvantage in education and the underlying mechanisms. To this end, we compare trends in $\hat{\upbeta}$ and Corr(*x*, *y*).

## Data and Variables

### Data

We compiled a new database—the International Sibsize and Educational Attainment Database (ISEAD)—that includes 111 national surveys from 26 countries. Countries and surveys were selected based on the following criteria. First, we focused on countries with low fertility during the 1990s, when the youngest cohorts in our samples were of school-attending age. We define *low fertility* broadly as below the replacement level of 2.1. This definition includes most industrialized countries in Europe, North America, Australia, and East Asia as well as many post-communist countries. In so doing, we are primarily interested in what happens to sibsize gaps in education during periods of declining family size as well as related social and demographic transformations. We excluded countries in other regions (e.g., Latin America, South and Southeast Asia, and Africa) that were either less developed or developing during the 1990s.^2^ Second, surveys had to be nationally representative of the adult population. We excluded surveys of young children/adolescents because subjects were too young to have completed their formal education. Third, surveys had to contain information on respondents’ number of siblings,^3^ educational attainment, parental educational attainment (the highest of the father’s and mother’s education, or the father’s if mother’s education is unavailable), age or year of birth, and sex. Finally, we limited our sample to data collected from 1950 onward.

Table 2 lists 26 countries with surveys that satisfied our criteria. Section B of the online appendix provides a full list of surveys and more information on sample specification. Links to the relevant data archives and a replication package for the data and analysis are provided on the ISEAD website (https://dataverse.harvard.edu/dataverse/isead).

**Table 2:** List of countries and cohorts from the International Sibsize and Educational Attainment Database (ISEAD)

	1901–1910	1911–1920	1921–1930	1931–1940	1941–1950	1951–1960	1961–1970	1971–1980	1981–1990	Number of Cohorts	Region
Australia (AU)			●	●	●	●	●	●		6	ANS
Belgium (BE)				●	●	●	●	●		5	WEU
Bulgaria (BG)			●	●	●	●	●	●		6	EEU
Canada (CA)		●	●	●	●	●	●	●		7	ANS
China (CN)	●	●	●	●	●	●	●	●	●	9	EAS
Taiwan (TW)			●	●	●	●	●	●	●	7	EAS
Czech Republic (CZ)			●	●	●	●	●	●		6	CEE
Estonia (EE)			●	●	●	●	●	●		6	FSU
France (FR)		●	●	●	●	●	●	●		7	WEU
Georgia (GE)				●	●	●	●	●		5	FSU
East Germany (DE-E)			●	●	●	●	●	●		6	EEU
West Germany (DE-W)		●	●	●	●	●	●	●	●	8	WEU
Hungary (HU)			●	●	●	●	●	●		6	EEU
Italy (IT)				●	●	●	●	●		5	SEU
Japan (JP)			●	●	●	●	●	●	●	7	EAS
South Korea (KR)				●	●	●	●	●	●	6	EAS
Lithuania (LT)				●	●	●	●	●		5	FSU
Netherlands (NL)			●	●	●	●	●	●		6	WEU
Norway (NO)				●	●	●	●	●		5	NEU
Poland (PL)			●	●	●	●	●	●	●	7	CEE
Romania (RO)			●	●	●	●	●	●		6	EEU
Russia (RU)			●	●	●	●	●	●		6	FSU
Spain (ES)				●	●	●	●	●	●	6	SEU
Sweden (SE)				●	●	●	●	●	●	6	NEU
United Kingdom (GB)	●	●	●	●	●	●	●	●	●	9	ANS
United States (US)	●	●	●	●	●	●	●	●	●	9	ANS
Number of Countries	3	6	18	26	26	26	26	26	10	167	

*Note*: WEU = Western Europe; NEU = Northern Europe; SEU = Southern Europe; CEE = Central-Eastern Europe; EEU = Eastern Europe; FSU = Former Soviet Union; ANS = Anglo-Saxon; and EAS = East Asia.

To ensure that respondents had completed their formal education, we limited our samples to individuals aged 27 or older at the time of the survey. To take a cohort-based approach, we pooled the surveys by country and then broke each pooled national sample into nine 10-year birth cohorts covering the twentieth century: from those born between 1901 and 1910 to those born between 1981 and 1990. For a robust estimation, we used country-cohort samples with at least 500 observations.^4^ The final sample includes 536,124 respondents from 167 country-cohort samples. For each country, we cover at least five birth cohorts, from the cohort of 1931–1940 to the cohort of 1971–1980. We limit our analyses to this subset of birth cohorts whenever we intend to make direct cross-national comparisons.

### Variables

*Sibship size* is the total number of brothers and sisters a respondent has ever had, not including the respondent. Because many surveys did not specify whether respondents should count half-siblings or nonbiological siblings, such as step- and adopted siblings, we assumed that sibsize is inclusive of all types of siblings. To minimize the effect of arbitrary variation in the upper bound of sibsize across surveys, we capped sibsizes of 10 and more at 10.^5^

We measure *educational attainment* as the years of education completed. We chose this measure for its comparability across countries with wide variation in educational systems. Only a few surveys asked respondents about the actual number of years of school they completed. For parental education, this was even rarer. Most surveys instead measured education categorically (e.g., the highest level of educational level completed or the highest qualification attained). Although some surveys employed internationally standardized schemes (e.g., ISCED), most surveys used country-specific schemes that were not easily comparable with other countries.

To construct the educational measure as consistently as possible across our 26 countries, we used mapping schemes provided by UNESCO’s Institute of Statistics (http://uis.unesco.org/en/isced-mappings). These guides allowed us to harmonize country-specific educational schemes by providing the theoretical number of cumulative years corresponding to each level of schooling or qualification within a given country. Using these mappings, we constructed a measure of educational attainment capturing the theoretical years of schooling for a person with a certain level of schooling or qualification, rather than the actual length of a person’s schooling.^6^ We provide cohort trends in educational attainment for all 26 countries in Fig. C2 of the online appendix.

### Analytical Strategy

Our main aim is to describe how sibsize and educational disadvantage associated with an additional sibling have changed across birth cohorts for each of the 26 countries. We estimate the mean sibsize for each cohort-country sample conditional on gender, age, and survey effects. Likewise, we estimate the ordinary least squares (OLS) coefficient of sibsize on education using the following model specification:

2 $$ {education}_{itc}={\upbeta}_{0, tc}+{\upbeta}_{1, tc}{\left( number\ of\ siblings\right)}_{itc}+{\upbeta}_{2, tc}{(female)}_{itc}+{\upbeta}_{3, tc}{\left( ag e\right)}_{itc}+{\upbeta}_{4, tc}{\left( ag{e}^2\right)}_{itc}+{\upbeta}_{5, tc}{\left( ag{e}^3\right)}_{itc}+{\sum}_k{\upgamma}_{k, tc}{\left( surve{y}_k\right)}_{itc}+{\upvarepsilon}_{itc}, $$

where *i* denotes individual, *t* denotes birth cohort, *c* denotes country, and *k* denotes the survey marker identifying different surveys within a country-cohort sample. The parameter β_1, *tc*_ is our central interest. The general aim is to examine whether and how β_1, *tc*_ changed across birth cohorts and how this cohort change varies across countries.

## Results

### Cohort Trends in Sibsize

**Fig. 1:**
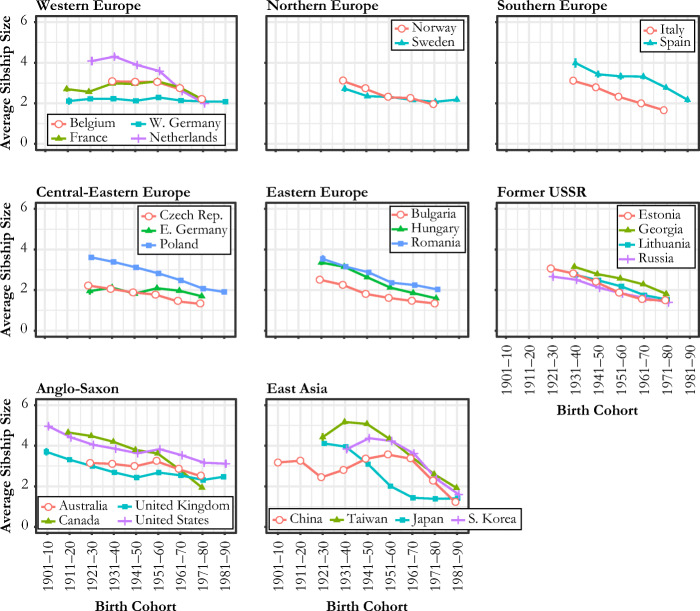
Cohort trends in average sibship size

Figure 1 shows the cohort trends of average sibsize over the past century in 26 countries. A common trend is that almost all countries experienced a decline in sibsize. However, there are a few notable patterns of variation between and within regions.

First, the changes are most dramatic in East Asian countries. For the cohort born between 1951 and 1960, South Koreans have about 4 siblings on average; however, for the most recent two cohorts (1971–1980 and 1981–1990), average sibsize dropped to around 2. A similarly sharp fall is observed for China. Chinese adults who were born after 1980 have an average of 1.5 siblings—a level substantially lower than in previous cohorts. Taiwan also started a sharp decline from the birth cohort of 1951–1960 onward. Although Japan shows a similarly rapid decline in sibsize, the decline began much earlier than in other countries.

A second notable trend is that Anglo-Saxon countries show relatively high average sibsizes even in the latest cohorts. The United States, United Kingdom, and Australia all show average sibsizes significantly higher than 2 in the 1981–1990 birth cohort; this is compared with countries in other regions, which typically dropped to average sibsizes of 2 or below by this time. Exceptions to this trend are the two most recent birth cohorts of Canada, particularly those born after 1960, who have considerably smaller sibsizes than their older counterparts. Canada was very similar to the United States in trends until the 1951–1960 cohort, but the two countries began to diverge thereafter: the 1970s cohort tended to have an average of about 2 siblings in Canada compared with 3 siblings in the United States. Another interesting pattern among Anglo-Saxon countries (with the exception of Canada) is a short-term increase in sibsize that corresponds to the Baby Boomer cohort.

European countries, despite varying intercepts and rates of decline, all appear to converge to about 2 siblings or just below in the most recent birth cohorts. The Netherlands and Spain show a particularly sharp fall, especially among the two most recent birth cohorts. In contrast, a more gradual downward or even flat trend can be observed among countries in other European regions. One interesting pattern is the flatness of the sibsize trend across birth cohorts in West Germany, which indicates that individuals have always had 2 siblings on average over the last century. Relative to Western and Northern European countries, Central-Eastern and Eastern European countries show lower sibsizes in the most recent cohorts, with an average of about 1.5 siblings, compared with 2 siblings elsewhere.

Figure 1 offers new evidence of how sibsize has changed across cohorts and how this change varies across countries. Sibsize provides a measure of family size from the child’s perspective. It captures aspects of family size that are distinct from those captured by the number of children (or fertility level), which is a measure of family size taken from the parents’ perspective (Fahey 2017; Preston 1976). Sibsize is the most appropriate indicator of family size if the life chances and well-being of children are the main interest (Fahey 2017). Despite this, there is surprisingly little evidence within the existing literature on patterns in sibsize across space and time, which is potentially due to a lack of harmonized, comparative data. Indeed, existing comparative evidence has either been drawn indirectly from fertility information (Shkolnikov et al. 2007) or from country-specific analyses that varied widely in their methods of defining and measuring target populations and key variables (Steelman et al. 2002). In this vein, the cohort trends in Fig. 1 should be interpreted as distinct from conventional trends in fertility. Sibsize trends reflect not only changes in the average number of children among parents but also changes in how the number of children is dispersed across families (Preston 1976; Shkolnikov et al. 2007). For example, West Germany’s flat trend in sibsize in Fig. 1 shows that the country’s falling fertility rate must be largely driven by the growing share of childless women rather than a decline in the number of children among mothers. This suggests that differences between average sibsize and fertility rate trends can be attributable to nonlinear distributional changes in fertility (see Fig. C1, online appendix, for fertility plots comparable to Fig. 1).

### Cohort Trends in the Sibsize Penalty in Educational Attainment

**Fig. 2:**
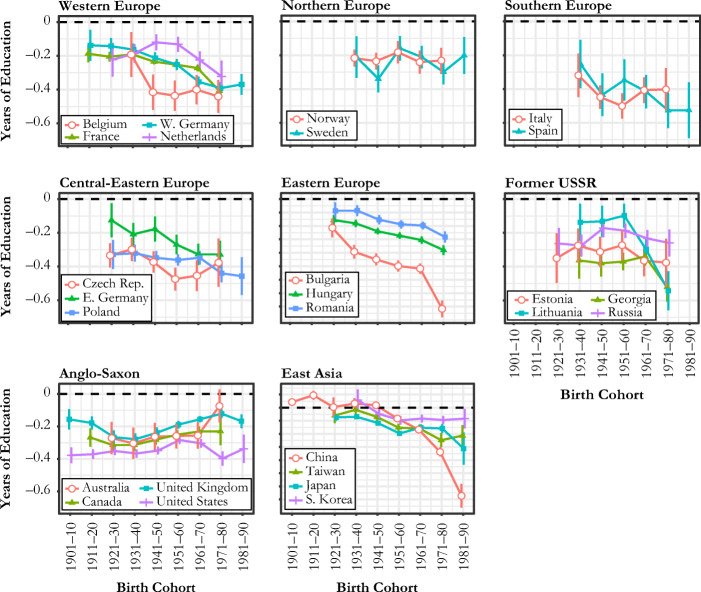
Cohort trends in sibship size disadvantage in the years of education: whiskers show 95% confidence intervals of the coefficient. The scale of *y*-axis is widely different for Eastern Europe and East Asia due to Bulgaria and China.

Figure 2 shows how the estimated coefficients of sibsize on the years of education, net of gender, age, and survey effects varied across cohorts in 26 countries. Negative values here indicate a disadvantage in educational attainment associated with having an additional sibling.

A first observation is that in all countries and for most birth cohorts, an additional sibling is associated with significantly fewer years of schooling, a pattern consistent with existing evidence on sibsize disadvantage in developed countries. For East Asians born before 1950, however, such a sibling penalty was not so evident: it was either substantively small (Japan and Taiwan) or not measurably different from 0 (China and South Korea). This is consistent with the argument that the effect of sibsize on educational attainment is sensitive to the context of economic development and may even be positive (Maralani 2008). In more recent cohorts, the East Asian coefficients became more negative and began to align with trends observed for Western countries.

When it comes to cohort trends, we do not find any evidence of a consistent decline in sibsize disadvantage across countries. This result does not support the prediction highlighting the role of expanding public policies for moderating the sibsize penalty during the development of the modern welfare state (H1). In contrast, sibsize disadvantage in education appears to have increased in most regions, although the magnitude of this increase varies across regions and the trends show volatile fluctuations in some countries. This increase is most prominent in Western European, post-communist, and East Asian countries, despite notable intraregion variation.

In Western Europe (panel a in Fig. 2), we see that all four countries show notable increases in the sibsize penalty after the birth cohort of 1931–1940. For example, for those born in 1930s in all four countries, having an additional sibling was associated with a roughly 2.4-month reduction in schooling. However, for those born in the 1970s, this penalty grew to around 3.6 months in the Netherlands and to about 5 months in France, West Germany, and Belgium. A similar trend is observed for Spain (panel c). For the two Nordic countries (Norway and Sweden; panel b) as well as Italy (panel c), the trends are not as directional: trends in these countries seem to fluctuate unpredictably, at around –0.2 and –0.3 for Norway and Sweden and around –0.4 for Italy.^7^

European countries with communist legacies (see panels d, e, and f of Fig. 2) show growing sibsize penalties but with considerable variation. For example, having an additional sibling was associated with a 0.2-year reduction in educational attainment in East Germany for the 1930–1940 birth cohort, which grew to about 0.35 years for the 1960–1970 birth cohort. Steeper increases in the sibsize penalty are observed in Romania and Hungary: the penalty grew from 0.2–0.3 years for the 1920–1930 cohort to about 0.6–0.7 years for those born in 1970s. More outstanding still is Bulgaria, where the sibsize penalty grew from about 6 months of schooling for the 1921–1930 birth cohort to 1 year for those born in the 1960s and ultimately reached about 1.5 years for the 1971–1980 cohort. Growing sibsize disadvantage is common in the former Soviet Union republics (panel f), but patterns are nonlinear. Lithuania and Georgia show a substantial decline in the coefficient for recent birth cohorts, especially those born in the 1970s, where an additional sibling is associated with a more than half-year reduction in schooling. Estonia shows only a slight decline starting from –0.3 for the 1931–1940 cohort to –0.4 for the 1971–1980 cohort. A less clear trend is observed for Russia, where the coefficient shows a secular but nonsignificant decline starting with the 1941–1950 cohort. Russia has the smallest sibsize penalty in education among the four former Soviet Union countries in the most recent cohort.

Anglo-Saxon countries (panel g) present an exceptional case where no countries show a declining trend in the coefficient of sibsize on education. The United States shows a steadily upward trend that peaks for those born in 1950s and 1960s, followed by fluctuations among later-born cohorts, which is in line with a recent study on the U.S. experience (Gibbs et al. 2016). The other three countries show increasingly smaller disadvantage among recent birth cohorts, suggesting that the educational price of an additional sibling has reduced. For example, for those born in the 1930s, an additional sibling comes with a reduction of about 0.3 years in schooling in Australia, Canada, and the United Kingdom. For those born in the 1970s, however, this penalty reduces to about 0.1 years in Australia and the United Kingdom and to about 0.25 years in Canada.

All East Asian countries show significant downward trends. They share a gradual decline from near-zero coefficients among the earliest cohorts but began to diverge after the 1961–1970 cohort. China and Japan experienced the most dramatic increases in sibsize disadvantage, from 0.3 to 0.6 years in Japan and from 0.3 to 1.3 years in China. South Korea, on the other hand, shows a flat curve following the 1961–1970 cohort, fluctuating mildly between about a 0.15- to 0.2-year sibsize disadvantage. Taiwan shows a more negative coefficient than Korea in general, reaching a 0.4-year sibsize disadvantage in education in the most recent cohort. The trends observed for China and Taiwan are generally consistent with patterns documented by previous studies (Lu and Treiman 2008; Parish and Willis 1993).

**Fig. 3:**
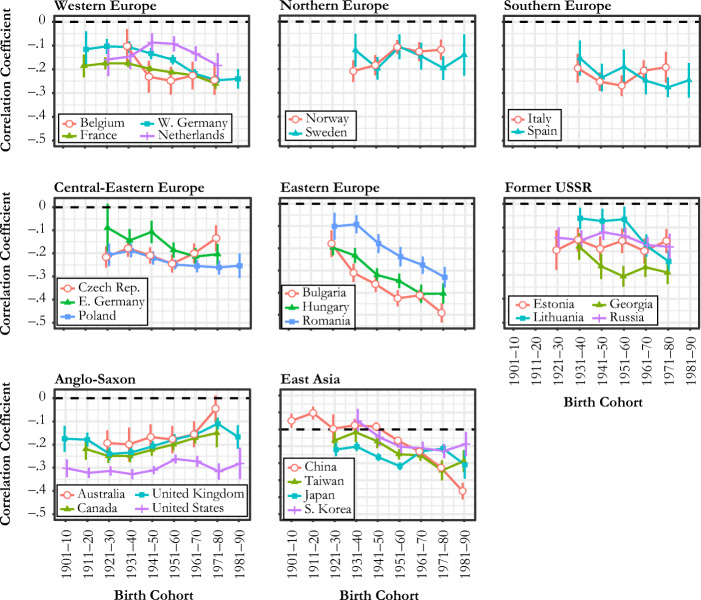
Cohort trends in the correlation coefficient between sibship size and the years of education: correlations are semipartial correlations between sibship size net of gender, age, and survey effects and educational attainment. Whiskers show 95% confidence intervals of the coefficient that are constructed from bootstrap standard errors. The scale of *y*-axis is slightly different for Eastern Europe and East Asia.

To disentangle the trends observed in Fig. 2 from the changes due to the compressed distributions of sibsize and educational attainment, we plot the cohort trends in the correlation coefficients between sibsize and educational attainment in Fig. 3. The trends in Fig. 3 are generally consistent with those in Fig. 2, but several countries—such as France, Spain, East Germany, Poland, and Japan—show much flatter trends than those shown in Fig. 2. More remarkable are Bulgaria and China: the dramatic plummets shown in Fig. 2 become much more moderate downward trends in Fig. 3 that are now comparable to other neighboring countries. This difference suggests that the sharp increases in educational disadvantage by sibsize in these countries are attributable to the widening dispersion of educational attainment and the compression of sibsize on top of a changing relationship between sibsize and education.^8^ On the other hand, Norway and the Czech Republic are two countries whose weakening negative correlations have been offset by distributional changes in education and sibsize.

**Fig. 4:**
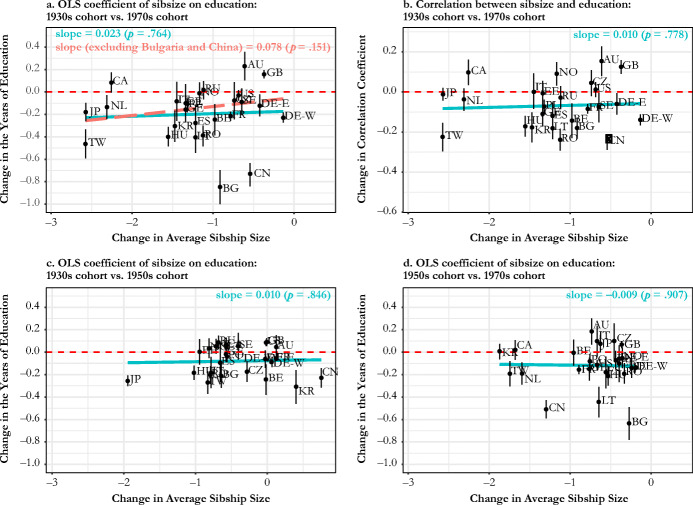
Changes in average sibship size and sibship size disadvantage in the years of education between the 1931–1940 cohort and the 1971–1980 cohort: in panel a, the blue fitted line is based on the whole country sample, and the red (dashed) fitted line is based on the country sample excluding China and Bulgaria as outliers. The whiskers show the 95% confidence intervals.

In Fig. 4, we show how countries vary by the magnitude of change in the relationship between sibsize and educational attainment between the 1931–1940 and 1971–1980 cohorts, paying particular attention to changes in average sibship size as an indicator of the changing family demographic context. We compare changes in the OLS coefficient (panel a) and the correlation coefficient to infer the role of distributional changes in sibsize and/or educational attainment (panel b). According to panel a, the sibsize-education penalty significantly decreased in only 2 of 26 countries (United Kingdom and Australia). In contrast, we see evidence of a significantly increasing sibsize-education penalty in 16 countries. Eight countries show either positive or negative changes that were not statistically significant. Looking to panel b, most countries moved up toward or over the line indicating zero association. The sibsize-education penalty now shows a significant reduction in two more countries (Canada and Norway), and 13 countries show significant increases in this penalty. Nine countries fail the reject the hypothesis of null change. The fitted lines in panels a and b show that the scale of change in family size context is not accompanied by the magnitude of changing sibsize disadvantage in education, regardless of whether we purge the influence of the distributional change in sibsize (as in panel b) or do not (as in panel a). Panels c and d show the results from additional analyses to check whether panel a conceals any meaningful patterns due to nonlinear cohort changes (e.g., Georgia and Lithuania). These panels show that the changes in the sibship penalty generally increased both across older cohorts (born in 1930s to born in 1950s) and across younger cohorts (born in 1950s to born in 1970s), but cross-country variation is more notable in the more recent cohorts. These panels also show that there is virtually no relationship between changes in sibsize disadvantage and changes in average sibsize.

To summarize, the results reported in Fig. 2 to Fig. 4 show that most countries experienced increasingly negative associations between sibsize and educational attainment throughout the past century, and only a minority of countries experienced weakening associations. Crucially, we also find considerable variation in these trends across countries. According to the result shown in panel a of Fig. 4, disadvantage in educational attainment associated with an additional sibling increased remarkably among post-socialist countries and East Asian countries by an average of 0.3 years and 0.34 years, respectively,^9^ and, to a lesser extent, Western European countries by 0.2 years. In contrast, there was no meaningful change in two Nordic countries (a 0.05-year increase), whereas Anglo-Saxon countries show a 0.11-year decrease on average.

### Explaining Cross-National Variation in the Cohort Trend of Sibsize Penalty

How can we understand our findings in relation to the hypotheses advanced earlier? The results shown in Figs. 3 and 4 strongly suggest that important regional factors are at play.

First, no Anglo-Saxon countries show significant downward shifts in the coefficient of sibsize on schooling between birth cohorts, with three (Australia, Canada, and the United Kingdom) in fact showing an upward shift. Both Nordic countries (Norway and Sweden) also show no changes in sibsize disadvantage. Given that we have only two Nordic countries, however, it may be premature to attribute this trend to the unique institutional characteristics shared by these countries. However, our findings suggest that Anglo-Saxon and Nordic countries may deviate from a more general trend of increasing sibsize disadvantage in education throughout the rest of Europe and East Asia. Changes in public policies may partially explain this cross-national variation (H1). For example, the absence of notable changes in Sweden and Norway across cohorts, as well as their comparatively small sibsize disadvantages from the outset, may be indicative of the strong buffering effects of their exceptionally inclusive social policies (Park 2008; Xu 2008).

Second, we find that 8 of 11 countries with communist legacies, including China, underwent increasing sibsize disadvantages, especially among the most recent cohorts who experienced the post-communism period as adolescents. This provides support for our second hypothesis (H2). As Lu and Treiman (2008) inferred from their Chinese case, the rapid transition to a market-based economy may have contributed to the strengthened negative associations between sibsize and educational attainment observed over time.^10^ Recent comparative evidence of declining intergenerational mobility after the post-communist transition among Central-Eastern and Eastern European countries (Jackson and Evans 2017) and earlier findings for Russia (Gerber 2000; Gerber and Hout 2004) also corroborate our findings.

**Fig. 5:**
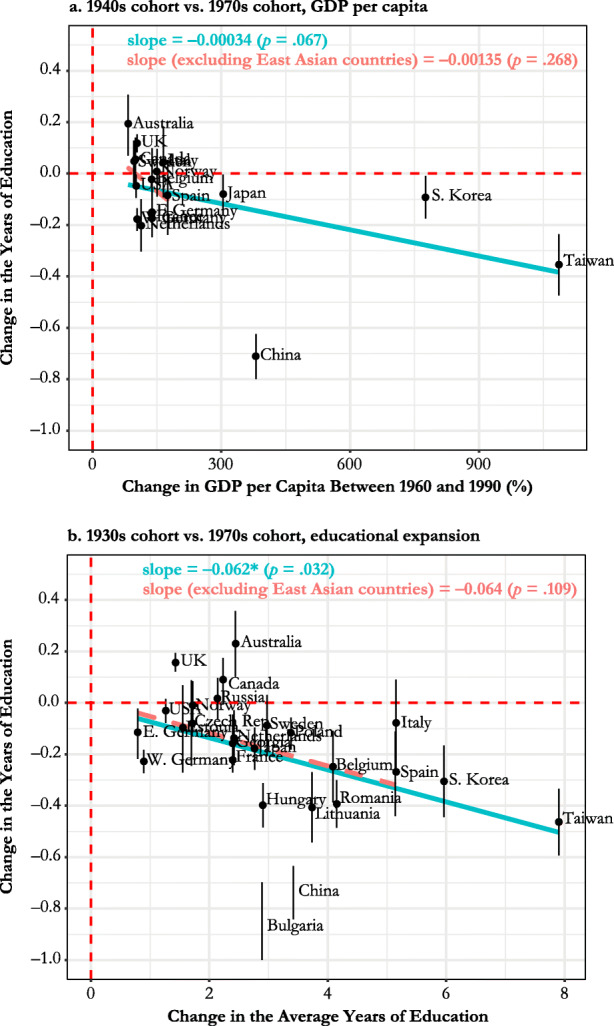
Economic growth, educational expansion and cohort changes in sibship size disadvantage in the years of education: the red dashed line shows the fitted line when East Asian countries are excluded. GDP per capita is are measured by the three-year averages around the beginning year (1960) and the end year (1990). These years, 1960 and 1990, capture the teenage years of the 1941–1950 cohort and the 1971–1980 cohort. We compare the 1941–1950 cohort (not the 1931–1940 cohort) with the 1971–1980 cohort because GDP per capita is available only from 1960 for many countries. For educational expansion, we use the difference in the average years of educational attainment between the 1931–1940 cohort and the 1971–1980 cohort from our ISEAD samples. *Source:* World Bank database (GDP per capita).

Third, we find growing sibsize disadvantages in East Asian countries. This is largely consistent with our third prediction (H3), which highlights the importance of rapidly changing education and labor market institutions over the course of accelerated economic development. East Asian countries experienced radical structural changes in education and industry both in absolute and in relative terms, and exhibited the most pronounced quantity-quality transition (i.e., rapidly expanding education and rapidly declining family size) throughout the second half of the past century (Montgomery et al. 2000; Parish and Willis 1993). High anxiety among potential parents about their future children’s educational success suppresses fertility (Anderson and Kohler 2013), illustrating one important aspect of the sharp quantity-quality transition in East Asian societies. Fig. 5 presents results from additional analyses examining whether cohort changes in sibsize disadvantage across countries are explained by their patterns of economic growth (panel a) and educational expansion (panel b). For economic growth, we use change in GDP per capita between 1960 and 1990, which captures the teenage years of the 1940s cohort and the 1970s cohort.^11^ For educational expansion, we use change in the average years of educational attainment between the 1930s cohort and the 1970s cohort from our ISEAD samples. Panels a and b show that exceptional economic growth and educational expansion in East Asian countries co-occurred with growing sibsize disadvantage in educational attainment but that such a relationship is not so evident among other countries.^12^

### Role of Parental Education

To examine the contribution of differential family size to the trends in the sibsize-education penalty, we explore whether a growing gap in children’s educational attainment between small and large families is due to parental SES becoming an increasingly strong predictor of family size. To test this, we use parental education as an indicator of parents’ SES and quantify how much of the educational disadvantage associated with sibsize shrinks when parental education is controlled for and how much remains.^13^ This analysis is one way to gauge how intergenerational mobility in educational attainment operates through sibsize.^14^

**Fig. 6:**
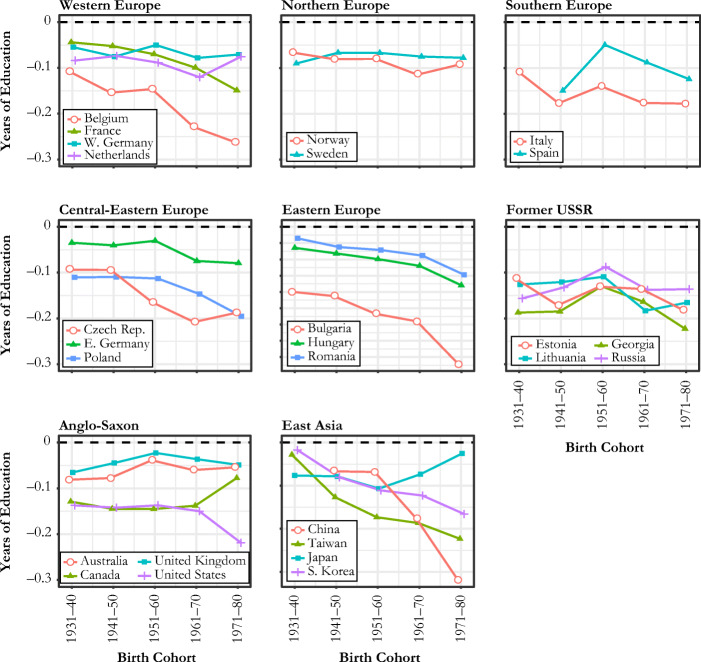
Trends in sibship size disadvantage in the years of education explained by parental education from the 1931–1940 cohort to 1971–1980 cohort. The scale of *y*-axis is different for Eastern Europe due to Bulgaria.

In Fig. 6, we plot how much of the sibsize coefficient on education is explained by parental education and how this varies across cohorts. The *y*-axis presents the difference between the sibsize coefficients before and after controlling for parental education, which indicates the confounding role of parental education. The results show that the size of sibsize coefficient increased for all birth cohorts in all countries, highlighting the important role of differential family size by parental education in explaining sibsize gaps in educational attainment. Our calculations show a roughly 40% reduction in the sibsize coefficient on average when parental education is controlled for (see Fig. C5 in the online appendix). However, we also see substantial regional and country-level variation. In many countries, especially those in Central-Eastern and Eastern Europe, some in Western Europe (Belgium and France), those in East Asia (except Japan), and the United States, differential family size by parental education explains an increasingly larger fraction of the sibsize penalty among later-born cohorts. Other countries show generally flat or fluctuating changes with notable cross-national variation in the magnitude (i.e., intercepts): East/West Germany, the Netherlands, Norway, Sweden, Australia, and the United Kingdom show estimates of the role of parental SES below 0.1 years, while others such as Italy, Spain, and former USSR republics show relatively high estimates. Two exceptional countries, Canada and Japan, show a weakening role of parental SES observed among the youngest cohorts. However, in general, findings suggest a strengthening or at least stable role of differential family size by parental SES across cohorts.

**Fig. 7:**
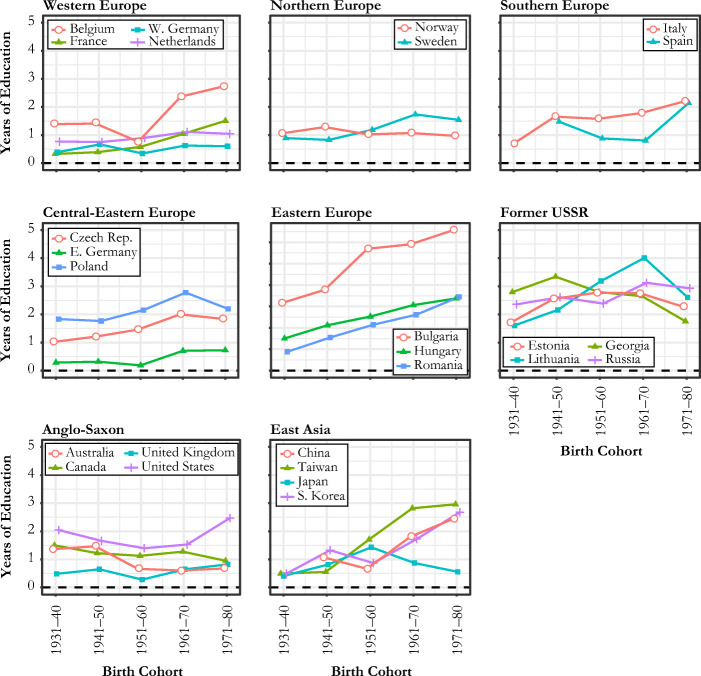
Trends in gap in parental education between children with small sibship sizes (0–1) and children with large sibship sizes (3+) from the 1931–1940 cohort to the 1971–1980 cohort. The scale of *y*-axis is different for Eastern Europe due to Bulgaria.

Figure 7 corroborates this inference. The figure shows the cohort trends for the gap in parental education between children with small sibsize (0 or 1) and those with large sibsize (3 or more). The pattern largely mirrors that in Fig. 6: countries with increasing gaps in parental SES by sibsize are also those showing an increasing role of parental SES in the sibsize-education penalty and vice versa. For example, in Belgium and France, two countries where the sibsize gap by parental education has been widening, the role of parents’ SES in explaining sibsize disadvantage has increased substantially. Japan, on the other hand, shows a reduction in both the sibsize gap by parental education as well as the role of parental education in explaining the sibsize penalty. This remarkable similarity between Fig. 6 and Fig. 7 highlights the importance of differential family size during the demographic transition as a key mechanism underlying the intergenerational transfer of educational attainment and provides empirical support for H4.^15^

## Discussion

We present evidence from a new comparative database on how sibship size and sibship size disadvantage in education have changed across birth cohorts over the past century. Our analyses reveal some general cohort trends as well as notable variations across countries. First, a central finding of our study is the overall trend of growing sibsize disadvantage. In the majority of the countries studied, the sibsize penalty in educational attainment became stronger. Between the 1931–1940 birth cohort and the 1971–1980 birth cohort, 16 of 26 countries showed a statistically significant increase in the sibsize disadvantage in education, and only 2 countries showed a significant reduction. Second, these trends vary substantially between and within regions. The increasing trend of sibsize disadvantage is strongest among post-socialist (0.3 years) and East Asian countries (0.34 years) and, to lesser extent, in Western Europe (0.2 years). In contrast, the trend of growing sibsize disadvantage is absent in Nordic countries (0.05 years) and is either absent or reversed in Anglo-Saxon countries (–0.11 years, on average).

We evaluate these findings against the predictions made in H1–H4. In Table 3, we juxtapose these hypotheses (Table 2) against the findings of this study (Fig. 2 and Fig. 7). We argue that our hypotheses on the role of public policies and the transition of economic systems offer reasonable explanations for the increasing trends observed in post-communist and East Asian countries, as well as the persistently weak trends seen in the two Nordic countries. In addition, growing differential family size provides a convincing account of the strengthened trends in sibsize disadvantage in several countries across different regions, including some in Western Europe (e.g., France and Belgium).

**Table 3:** Mapping the findings of cohort trends in sibsize disadvantage to hypotheses across regions

	Public Policies				
	Inclusive Social and Welfare Policies	Transition From Socialist to Market Economy	Rapid Restructuring in Markets and Education	Growing SES Gap in Family Size (Fig. 7)	Findings (Fig. 2)
Western Europe	–			+ (Belgium, France)	+
Northern Europe	–				
Southern Europe			+	+	+ (Spain)
Central-Eastern and Eastern Europe		+		+	+
Former USSR		+		+ (Lithuania)	+ (Georgia, Lithuania)
Anglo-Saxon					–
East Asia		+ (China)	+	+ (excluding Japan)	+

*Note*: – indicates weakening sibsize disadvantage; + indicates strengthening sibsize disadvantage.

Nonetheless, we highlight several regional and country-specific trends that do not fit well with the proposed accounts. For example, the four Western European countries show generally increasing levels sibsize disadvantage despite their relatively strong and increasingly inclusive public policies. Growing differential family size is also a plausible explanation for some of these Western European countries. Our hypotheses also predict a growing sibsize penalty in Southern Europe, and we find such a trend in Spain but no convincing evidence in Italy. There is also notable intraregion variation that is not captured by our macro-level explanations, such as the variation among former USSR republics as well as the notable divergences among East Asian countries in later-born cohorts. Elaborating detailed explanations for these unexplained patterns would require a focus on more nuanced country-specific factors, which is beyond the scope of this study. As such, a considerable amount of the cross-national and temporal variation in sibsize disadvantage remains unexplained by our proposed hypotheses.

Perhaps the most remarkable finding that remains largely explained in Table 3 is the pattern of stagnant or weakening sibsize penalty among Anglo-Saxon countries. Anglo-Saxon countries by and large have not undergone the same types of wide-scale changes in the macro-level factors featured in our hypotheses that have occurred in other countries. Thus, a reasonable prediction from the absence of these factors would be a relatively stable trend. However, we see patterns of weakening sibsize disadvantage. Despite a recent study indicating that the weakening negative sibsize effect on education in the United States “may be the result of the expanding size of state-sponsored investments in education and related programs that unlink family background from educational opportunities” (Gibbs et al. 2016:738), our cross-national comparison suggests that an interpretation focusing exclusively on the role of public policies may be too general and would need to be supplemented by additional institutional and cultural factors shared by other Anglo-Saxon countries. Indeed, Anglo-Saxon countries produce relatively high fertility despite what their liberal regime (e.g., weak public policies and more marketized approaches) would predict (McDonald and Moyle 2010), highlighting the importance of other historical and cultural factors, such as religiosity, a pro-child value orientation, and large immigrant populations (Hayford and Morgan 2008; McDonald and Moyle 2010; Morgan 2015; Sigle 2016). For example, individuals in the United States and United Kingdom tend to feel that having a child is less costly than other European counterparts (DiPrete et al. 2003). This suggests that the unique Anglo-Saxon pattern of weakening sibsize disadvantage may be related to persistently high fertility and the sociocultural factors sustaining it.

We believe that our study contributes to research on the role of family configuration in the intergenerational reproduction of educational attainment or, more generally, the attainment of SES, by providing cross-national evidence, which has been relatively rare because of data limitations. Our findings suggest that the universal demographic trend of reducing family sizes engenders two offsetting consequences for children. On one hand, such a compositional change makes fewer children suffer the penalties related to large family sizes, thereby reducing the importance of differential family size as a route to intergenerational transmission of SES (Fahey 2017; Präg et al. 2020). On the other hand, an increase in the negative coefficient of sibsize on education suggests an increasing penalty from growing up in large families, thereby boosting the importance of being born in a small family for achieving high educational attainment. Which one of these two forces supersedes the other remains an open question. Nonetheless, our study suggests that several macro-level changes in social and economic institutions and policies may moderate or mediate such intergenerational processes, thereby producing substantial cross-country variation.

In addition, growing levels of differential family size in terms of parental education in many countries (see Fig. 7) imply that changes in family size can be an important route for the intergenerational reproduction of education. Past research has shown that the number of children (from the parents’ perspective) is an important mediator in educational reproduction (Lawrence and Breen 2016; Maralani 2013; Mare and Maralani 2006). Shrinking family sizes during demographic transitions do not occur uniformly across parental SES groups. If the SES gradient in family size becomes increasingly negative (e.g., fewer children from high-SES parents) and the benefits of small family size become greater, potential parents opt to limit their family size as a strategy to pass advantages to their offspring. Our findings of remarkably increasing sibsize disadvantage in post-communist and East Asian countries indeed suggest that such a strategy has been adopted disproportionately among high-SES groups. This indicates that trends toward very small family sizes in these countries should be understood in relation to how intergenerational mobility operates and is perceived.

In this vein, our study calls for more comparative research on the role of demographic changes in the intergenerational transmission of socioeconomic (dis)advantages. Recent studies highlighting the importance of differences in marriage and fertility in intergenerational mobility (Kye and Mare 2012; Lawrence and Breen 2016; Maralani 2013; Mare and Maralani 2006; Song and Mare 2015) have largely focused on individual country cases, resulting in a scarcity of cross-societal and historical evidence. Yet, among low-fertility societies, new patterns of social stratification in marriage and fertility have been emerging over the past couple of decades. For example, in some countries, a positive relationship between fertility and female labor market participation can now be observed, suggesting that the economic empowerment of women is becoming an increasingly important predictor of country-level fertility (Myrskylä et al. 2009). However, such stratifying patterns of family formation have not emerged uniformly across societies; rather, they have depended largely on country-specific institutions and policies. Women’s empowerment, for instance, is a complex concept that captures women’s relative well-being and life chances across multiple life domains. Although some countries have been successful in assembling the structural conditions necessary to empower women beyond success in schooling, others have failed to overcome the conventional traps due to insufficient policy support. This suggests that the role of demographic factors in the intergenerational transmission of resources and status may often vary predictably rather than randomly across societies. Our comparative study sheds light on one example of such processes.

An important limitation of our study is its focus on low-fertility countries at the turn of the past century, which may constrain the scope and generalizability of findings. For example, our study does not provide comparisons between “once high-fertility and now low-fertility” countries (e.g., East Asian countries) and “once high-fertility and still mid- to high-fertility” countries (e.g., many less-developed countries). One might infer that high-fertility countries would experience similar growth in the sibsize penalty if they underwent the same patterns of economic and institutional change as countries observed in this study, but such a generalization would ignore widely varying institutional and structural contexts of industrialization and development across regions and across countries. Indeed, it is noteworthy that substantial uncertainties remain across countries even within our relatively homogenous sample of low-fertility societies.

## Electronic Supplementary Material

ESM 1 (PDF 4351 kb)

## Data Availability

The data set generated and analyzed during the current study is available as the International Sibsize and Educational Attainment Database (ISEAD) on Dataverse: https://dataverse.harvard.edu/dataverse/isead.

## Footnotes

1 Sibsize (*x*) is a count variable, the mean of which is linked to its variance proportionally, and a decline in mean sibsize comes with a reduction in its variance. This means that, *ceteris paribus*, a simple reduction in average sibsize can cause a stronger negative coefficient of sibsize on educational attainment. We provide a formal account of this in section A of the online appendix. We can draw a similar inference about educational attainment, *y*. Educational expansion, which implies an increase in the average level of educational attainment, also implies that more people in the population get higher levels of education (Shavit and Blossfeld 1993) and that institutionalized schooling becomes an increasingly central component of individuals’ lives (Mayer and Schoepflin 1989) and the modern state (Meyer et al. 1992). In other words, it indicates a more compressed distribution in *y* and therefore a smaller SD(*y*).

2 We included East Asian and post-communist countries, which transitioned to low fertility status relatively recently. According to Fig. C1 in the online appendix, where we plot the trends of total fertility rate, all countries in our sample (except Estonia) were higher than the replacement level of fertility before 1950s and ended up near or below the replacement level in the 1990s, varying only by the time at which subreplacement occurred.

3 We excluded surveys containing incomplete information on sibsize, such as household-based surveys that provided information only on siblings living in the same household.

4 We included one country-cohort group with 495 observations: the 1931–1940 cohort of Spain.

5 Our results are not sensitive to this cutoff value.

6 An important limitation of our measurement strategy is that qualitative differences between educational systems are ignored. For example, many European countries have strict divisions even at the same secondary level in terms of how much a track is vocationally oriented. Years of schooling cannot accommodate such differences.

7 The trend of France in Fig. 2 differs from findings of Ferrari and Dalla-Zuanna (2010), who reported no notable trend in the sibsize effect for the country. They also reported a similarly stable trend in Italy, which is consistent with our findings here. We speculate that this difference comes from differences in the measurement of educational outcomes (dummy variables indicating attaining a high school degree and a college qualification rather than years of schooling), differences in statistical modeling (logistic rather than OLS), and different data sources.

8 The dispersion of education narrowed in most of countries as education expanded. China is the only exceptional country showing the growing dispersion of education, seemingly because China is still lower in terms of the average level of education compared with other countries. Bulgaria, Estonia, Georgia, Lithuania, and Romania—all of which experienced rapid post-socialist transitions in the 1990s—also show a notable increase in educational dispersion for the youngest cohort. See Fig. C3 in the online appendix for the trends of dispersions in education and sibsize.

9 In this calculation, we include China in the post-socialist countries but not in East Asian countries.

10 Another noteworthy policy source impacting family size and its stratifying consequences in China is the one-child policy (OCP). Despite the near-universal enforcement of this policy, many poor families in rural areas were allowed to have two children (Baochang et al. 2007), possibly contributing to a widening nationwide family SES gap in sibsize. However, the role of the OCP in explaining the long-term cohort trend in our analysis seems to be limited because the policy was introduced nationally in 1980 and mostly affected the youngest cohort only. Our results suggest that the OCP reinforced rather than triggered the trend of increasing sibsize disadvantage in education.

11 We compare the 1940s cohort—not the 1930s cohort—with the 1970s cohort because GDP per capita is available only from 1960 for most countries.

12 We also examine whether cohort change in sibsize disadvantage in education is related to changes in economic inequality and educational inequality. We report the results in Fig. D4 in the online appendix. We find no meaningful cross-national patterns explained by changes in economic and educational inequalities from our analyses, but we should be cautious in generalizing this finding to temporal changes within individual countries. For example, Gibbs et al. (2016:740) documented that recent growing inequality in the United States may reverse the weakening trend of sibsize penalty.

13 Parental education does not capture all confounding factors related to parental characteristics, SES, family size, and children’s educational attainment. As such, our results (in Fig. 6) should be considered as showing lower-bound estimates of the confounding role of parental characteristics.

14 Parental education matters either because of differential family size or because of its impact on the pool of family resources available to children. The former explanation infers the causal effect of parental education on family size, whereas the latter suggests parental education acts as a confounder influencing both one’s educational attainment and unmeasured family resources. In the present analysis, we are unable to disentangle these two mechanisms. Therefore, our evidence is associational rather than causal.

15 We carried out the same analyses with the relative measures of education and found substantively similar results. These results are summarized in Figs. C6–C8 in the online appendix.
