# Supplementary material for: Cohort Trends in the Association Between Sibship Size and Educational Attainment in 26 Low-Fertility Countries
Source: Demography. 2020 Jun 22;57(3):1035–62. doi: 10.1007/s13524-020-00885-5 (PMC7329769; doi:10.1007/s13524-020-00885-5)
Supplement: Supplementary file 1 — (PDF 4351 kb) [file 13524_2020_885_MOESM1_ESM.pdf]

Cohort Trends in the Association between Sibship Size and Educational Attainment in 26 Low-Fertility Countries

Online Appendix

Seongsoo Choi

(Yonsei University, South Korea)

Riley Taiji

(University of Oxford, Nuffield College, UK)

Manting Chen

(University of Oxford, UK)

Christiaan Monden

(University of Oxford, Nuffield College, UK)

This research received funding from the European Research Council (ERC) under the European Union's Horizon 2020 research and innovation programme under grant agreement No 681546 (FAMSIZEMATTERS).

A: A Formal Demonstration of How a Change in Sibship Size leads to a Change in the Coefficient of Sibship Size on Educational Attainment

The regression coefficients of sibship size on educational attainment for the first cohort (c1) and the second cohort (c2) denote the educational disadvantage of an additional sibling. Their covariance and variance can be expressed as

$$\hat{\beta}_{\mathrm{c1}} = \frac{\mathrm{Cov}(x_{c1}, y_{c1})}{\mathrm{Var}(x_{c1})}$$

$$\hat{\beta}_{\mathrm{c2}} = \frac{\mathrm{Cov}(x_{c2}, y_{c2})}{\mathrm{Var}(x_{c2})}$$

where x denotes the number of siblings and y denotes years of education. Since the number of siblings is a count variable and typically follows a negative binomial distribution, a decline in the average sibship size between two cohorts indicates a decline in the variance of sibship size and a possible extra reduction in variance due to a corresponding reduction in the over-dispersion parameter.

We therefore make three assumptions:

(1) Sibship size follows the negative binomial distribution with the over-dispersion parameter α, so that sibship size has mean μ and variance $\mu(1 + \alpha\mu)$. When $\alpha = 0$, the distribution reduces to the Poisson distribution, where the mean and variance are identical. As α gets larger, the variance becomes larger than the mean, indicating over-dispersion.

(2) There is no change in either the association between sibship size and educational attainment or in the association between sibship size and an unobserved confounder (call this z), which is associated with both sibship size and educational attainment across c1 and c2. That is, the change in average sibship size by a factor of θ ($0<\theta<1$) equally applies to all individuals such that

$$\mu_{x_{c2}} = \theta\mu_{x_{c1}},$$

$$x_{c2} = \theta x_{c1}$$

and

$$\mathrm{Corr}(x_{c1}, y_{c1}) = \mathrm{Corr}(x_{c2}, y_{c2})$$

(3) There is no change in the distribution of educational attainment between c1 and c2. That is, y_{c1} and y_{c2} are largely exchangeable in our derivation. Thus, we can express $\hat{\beta}_{c2}$ as

$$\hat{\beta}_{c2} = \frac{1}{\theta}\hat{\beta}_{c1}$$

given that the numerator $cov(x_{c1}, y_{c1})$ changes by a factor of θ and the denominator $var(x_{c1})$ reduces by a factor θ^2 as the mean and the over-dispersion parameter reduces as follows

$$\mu_{x_{c2}} = \theta\mu_{x_{c1}},$$

$$\alpha_{x_{c2}} = \theta^2\alpha_{x_{c1}}$$

Assumption (1) is empirically supported by our data. Assumption (2) is a very strong assumption that hardly fits the reality. But this extreme hypothetical scenario provides a good reference situation against which we can consider endogenous change between sibship size and the coefficient only, ruling out any possible involvement of exogenous sources of changes. In our data, several countries (e.g., Japan, the Netherlands, Italy, Estonia, Russia, Czech Republic and the US) show little change in the correlation between sibsize and educational attainment. That is, $\mathrm{Corr}(x_{c1}, y_{c1}) = \mathrm{Corr}(x_{c2}, y_{c2})$ between the cohort born in 1930s and the cohort born in 1970s (see (2) of Figure 4 in the manuscript). Japan and the Netherlands, amongst others, show significant declines in the coefficient of sibship size on education between the same cohorts (see (1) of Figure 4 in the manuscript). This suggests that their declines in the coefficient of sibship size on education are largely due to change in (SD(y)/SD(x)). They are also two countries with the most notable declines in μ_x, which implies a change in μ_x is closely linked to a change in SD(x) as a count variable. Assumption (3) can be easily achieved by using the standardized measure of educational attainment.

By assuming (1), (2), and (3), then, we can conclude that a simple reduction in sibship size by a factor of θ induces a change in the coefficient by a factor $1/\theta$.

B: Issues in Sample Specification

1. Survey Sample Selection

We aimed to identify all relevant surveys by following four steps. First, we searched the following data archives for relevant surveys: ICPSR (https://www.icpsr.umich.edu/icpsrweb/ICPSR/), CESSDA (http://cessda.net/), and GESIS (http://www.gesis.org/en/services/data-analysis/data-archive-service/). Next, for each survey in the International Stratification and Mobility File (a collection by Harry Ganzeboom, Donald Treiman, and Elizabeth Stephenson, http://www.harryganzeboom.nl/ismf/index.htm), we checked whether the survey included information on sibship size. Third, we checked large international survey programmes such as ISSP, ESS, SHARE, FFS, GGP, and EASS. Fourth, we tracked references in cross-nationally comparative social mobility studies, in particular Social Mobility in Europe (Breen 2004). Fifth, for countries where we could not identify surveys in the first four steps, we searched national data archives if available. Finally, for pragmatic reasons and reproducibility, surveys had to be accessible for free for academic use without restrictions on nationality or institutional affiliation. This means that we had to exclude registry data and secure access data.

Most of our surveys are cross-sectional because we intended to utilize as many data sources as possible to spot cross-national variations and cohort trends. The bottom line in our data search was that a survey had be nationally representative. Some older surveys target only a single gender. For example, the Oxford Social Mobility Inquiry in the UK (only males) and the Growth of American Families in the US (only females). However, they are still designed to be nationally representative for the given genders sampled. Our data include several household panel surveys (e.g., China Family Panel Study, German Socio-Economic Panel, UK Household Longitudinal Study, Panel Study of Chinese Family Dynamics in Taiwan). In those cases, we constructed cross-sectional representative samples from the multiple waves that offer the requisite variables. Our use of cross-sectional surveys is not ideal to examine cohort trends, but it was inevitable given that cohort-based surveys are rarely comparative and are largely concentrated in only a small number of countries.

We did not apply weights because our research design did not allow us to use sampling weights that many of surveys offered. Instead, we double-checked whether cohort trends are sensitive to the merge of multiple surveys that vary in sample size by examining whether cohort trends from different surveys show consistency in several selective countries, such as Germany, France, Japan, China and the UK. We found there was no discernable differences in cohort change across surveys within each of these countries.

2. Age Restriction

As described in the manuscript, we limited our samples to individuals who were at least 27 years old at the time of the survey to make sure that respondents had completed formal education. The results of our analyses are robust to a change in this age cutoff (e.g., 25 and 30). For several surveys that only asked about respondents' siblings who were older than age 18, we used a higher age cutoff (age > 35) assuming that no respondents had a sibling that was more than 18 years their junior. In one survey (UK Household Longitudinal Study; the only available survey with the information on parental education for the young cohorts of UK), respondents are asked to report the number of siblings alive. We include only young adults from this survey based on the assumption that young adults do not have siblings who are deceased.

3. Observations Missing on Parental Education

Out of total 536,124 individuals with sibship size and other required information from 26 countries and 166 country-cohort samples, 82,951 individuals are missing information on their parent's education. This is about 15.5% of the total sample.

25.8% of missing observations (21,381 individuals) are from China, 10.5% (8,750 individuals) are from Japan, and 28.8% (23,856 individuals) are from the UK. Missingness in these three countries is mostly due to their survey designs (e.g., parental education was not asked) rather than due to potentially bias-inducing non-response. For example, Chinese surveys covering old birth cohorts (from 1901-10 to 1931-40) did not ask parental education. Similarly, some UK surveys covering recent cohorts (from the 1941-50 cohort on) did not contain information about parental education by design.

Only about 35% of total missing observations are from the remaining 23 countries. They are mostly conventional missing cases (e.g., non-responses, don't know, etc.). Four country-cohort cells become missing as a whole because they fail to keep the minimum 500 observations after dropping observations missing on parental education. These are the oldest cohorts in Australia, East Germany, West Germany and Spain. We, however, find that these missing cases do not induce bias. See Fig.2 as well as Fig.B1 in this appendix.

Table B1 shows the numbers of observations for individual country-cohort cells with and without parental education.

Table B1 Numbers of observations for country-cohort cells and information on missing observations

	Model	1901 -1910	1911 -1920	1921 -1930	1931 -1940	1941 -1950	1951 -1960	1961 -1970	1971 -1980	1981 -1990	Total	Missing (%)	Missing % Total
Australia	(1)			711	863	1,205	1,342	1,510	964		6,595	1,703	
	(2)				582	855	1,164	1,380	911		4,892	(25.8)	2.1
Belgium	(1)				754	1,064	1,396	1,503	1,222		5,939	439	
	(2)				704	973	1,292	1,382	1,149		5,500	(7.4)	0.5
Bulgaria	(1)			1,010	2,159	2,641	2,642	3,393	2,010		13,855	462	
	(2)			951	2,076	2,557	2,571	3,294	1,944		13,393	(3.3)	0.6
Canada	(1)		1,726	3,381	4,110	6,063	7,395	7,024	1,200		30,899	1,234	
	(2)		1,649	3,151	3,826	5,674	7,171	6,999	1,195		29,665	(4.0)	1.5
China	(1)	3,051	2,806	565	2,641	5,370	7,395	8,832	6,406	1,370	38,436	21,381	
	(2)					1,233	3,015	5,989	5,572	1,246	17,055	(55.6)	25.8
Taiwan	(1)			783	1,424	2,225	4,209	3,258	1,295	733	13,927	393	
	(2)			744	1,354	2,129	4,092	3,228	1,269	718	13,534	(2.8)	0.5
Czech Republic	(1)			1,499	2,101	2,847	2,892	2,260	1,466		13,065	607	
	(2)			1,425	1,986	2,734	2,782	2,139	1,392		12,458	(4.6)	0.7
Estonia	(1)			549	1,184	1,252	1,465	1,403	1,080		6,933	11	
	(2)			547	1,179	1,251	1,463	1,402	1,080		6,922	(0.2)	0.0
France	(1)		1,399	5,877	13,074	22,401	24,112	14,554	6,193		87,610	1,676	
	(2)		1,383	5,731	12,773	22,042	23,702	14,260	6,043		85,934	(1.9)	2.0
Georgia	(1)				1,280	1,200	1,903	2,022	1,504		7,909	420	
	(2)				1,078	1,111	1,842	1,978	1,480		7,489	(5.3)	0.5
East Germany	(1)			501	1,450	1,564	2,076	1,933	1,385		8,909	1,031	
	(2)				1,360	1,453	1,931	1,829	1,305		7,878	(11.6)	1.2
West Germany	(1)		532	2,340	4,957	6,077	6,892	9,167	6,975	2,134	39,074	5,021	
	(2)			1,856	4,291	5,194	6,253	8,409	6,186	1,864	34,053	(12.8)	6.1
Hungary	(1)			1,519	2,741	3,604	3,072	2,549	2,034		16,149	825	
	(2)			1,362	2,545	3,241	3,627	2,527	2,022		15,324	(5.1)	1.0
Italy	(1)				595	2,221	2,199	2,467	1,036		8,518	200	
	(2)				556	2,146	2,151	2,434	1,031		8,318	(2.3)	0.2
Japan	(1)			2,280	6,150	9,336	8,555	8,625	6,572	548	42,066	8,750	
	(2)			1,753	4,961	7,519	6,564	6,476	5,537	506	33,316	(20.8)	10.5

Table B1 (continued)

	Model	1901 -1910	1911 -1920	1921 -1930	1931 -1940	1941 -1950	1951 -1960	1961 -1970	1971 -1980	1981 -1990	Total	Missing (%)	Missing % out of Total
South Korea	(1)				694	1,654	2,728	3,137	2,105	654	10,972	271	
	(2)				661	1,583	2,658	3,067	2,084	648	10,701	(2.5)	0.3
Lithuania	(1)				1,547	1,413	1,712	1,798	1,362		7,832	885	
	(2)				1,252	1,234	1,551	1,653	1,257		6,947	(11.3)	1.1
Netherlands	(1)			722	1,936	2,980	3,671	3,809	1,212		14,330	760	
	(2)			670	1,808	2,821	3,530	3,620	1,121		13,570	(5.3)	0.9
Norway	(1)				1,556	2,489	2,506	2,940	2,476		11,967	199	
	(2)				1,507	2,452	2,469	2,895	2,445		11,768	(1.7)	0.2
Poland	(1)			1,233	4,286	5,464	6,766	4,111	3,437	907	26,204	1,203	
	(2)			1,176	3,921	5,186	6,526	3,983	3,322	887	25,001	(4.6)	1.5
Romania	(1)			695	1,946	2,085	2,368	2,264	1,520		10,878	201	
	(2)			675	1,909	2,043	2,335	2,228	1,487		10,677	(1.8)	0.2
Russia	(1)			1,526	2,643	2,492	3,648	2,584	1,181		14,074	2,881	
	(2)			1,024	1,973	1,935	2,965	2,209	1,087		11,193	(20.5)	3.5
Spain	(1)				495	739	814	1,051	1,184	600	4,883	547	
	(2)					726	803	1,042	1,170	595	4,336	(11.2)	0.7
Sweden	(1)				763	1,726	1,631	1,782	1,591	660	8,153	573	
	(2)				713	1,622	1,515	1,648	1,466	616	7,580	(7.0)	0.7
United Kingdom	(1)	773	2,280	2,521	3,769	4,102	6,639	9,221	5,537	1,848	36,690	23,856	
	(2)	662	2,023	2,193	2,076	1,019	1,295	1,797	1,769		12,834	(65.0)	28.8
United States	(1)	2,325	5,348	7,064	6,699	9,663	9,406	6,184	2,901	667	50,257	7,422	
	(2)	1,542	3,380	4,960	5,945	8,985	8,808	5,799	2,774	642	42,835	(14.8)	8.9
**Total**	**(1)**	**6,149**	**14,091**	**34,776**	**71,817**	**103,877**	**120,064**	**109,381**	**65,848**	**10,121**	**536,124**	**82,951**	
	**(2)**	**2,204**	**8,435**	**28,218**	**61,036**	**89,718**	**104,075**	**93,667**	**58,098**	**7,722**	**453,173**	**(15.5)**	**100**

Notes: Model (1) shows the numbers of all observations with the information of sibship size and other required variables but not necessarily parental education. Model (2) shows the numbers of all observations with the information on sibship size, other required variables AND parental education.

Table B2 List of survey datasets with information on age/cohort, sex composition and missing observations

Country	Survey name	Survey year	% Female	Age range	Cohort range	No restriction	Restriction 1 (age 27 and over, birth cohorts 1901-1990)		Restriction 2 (non-missing on age, sex, educ., and sibsize)		Restriction 3 (non-missing on age, sex, educ., sibsize, and parent's educ.)	
						Sample size	Sample size	% Lost	Sample size	% Lost	Sample size	% Lost
Australia	International Social Survey Program	1986	51.3%	35-84	1902-1951	1,250	760	39.2%	742	2.4%	-	100.0%
	Generations and Gender Programme	2005	55.8%	27-98	1906-1979	7,125	6,120	14.1%	6,116	0.1%	5,406	11.6%
Belgium	Generations and Gender Programme	2008	52.1%	27-82	1928-1983	7,163	6,260	12.6%	6,219	0.7%	5,754	7.5%
Bulgaria	Social Stratification in Eastern Europe After 1989: General Population Survey	1993	52.1%	27-78	1915-1966	4,919	4,320	12.2%	3,862	10.4%	3,805	1.5%
	Generations and Gender Programme	2004	54.8%	27-85	1919-1977	12,815	10,515	17.9%	9,991	5.0%	9,585	4.1%
Canada	Canadian Class Structure and Class Consciousness Survey	1982	48.1%	27-80	1902-1955	2,577	1,982	23.1%	1,978	0.2%	896	54.7%
	Canadian General Social Survey	1995	55.5%	27-80	1915-1968	10,749	8,826	17.9%	8,549	3.1%	8,549	0.0%
	Canadian General Social Survey	2000	56.6%	27-80	1920-1973	24,310	20,388	16.1%	20,388	0.0%	20,228	0.8%
China	China Housing Survey	1993	39.8%	27-73	1925-1971	2,096	1,856	11.5%	1,730	6.8%	1,138	34.2%
	Chinese Longitudinal Healthy Longevity Survey	1998	51.2%	77-97	1901-1921	9,093	5,925	34.8%	5,820	1.8%	-	100.0%
	East Asian Social Survey	2006	55.0%	27-69	1937-1979	3,208	2,739	14.6%	2,683	2.0%	1,676	37.5%
	China Family Panel Studies	2010	51.4%	27-109	1901-1983	31,731	28,412	10.5%	28,203	0.7%	14,673	48.0%
Czech Rep	Social Stratification in Eastern Europe After 1989: General Population Survey	1993	54.2%	27-70	1923-1966	5,621	5,063	9.9%	5,013	1.0%	4,939	1.5%
	Generations and Gender Programme	2005	52.8%	27-79	1926-1978	10,006	8,188	18.2%	8,052	1.7%	7,519	6.6%
W Germany	International Social Survey Program	1986	53.4%	35-85	1901-1951	2,809	1,858	33.9%	1,798	3.2%	-	100.0%
	The German Socio-Economic Panel	1984-2015	52.5%	27-99	1901-1988	61,140	43,637	28.6%	30,892	29.2%	27,980	9.4%
	Generations and Gender Programme	2005	53.8%	27-80	1925-1978	7,760	6,771	12.7%	6,563	3.1%	6,374	2.9%
E Germany	The German Socio-Economic Panel	1990-2015	52.8%	27-95	1902-1988	14,730	10,544	28.4%	7,917	24.9%	7,400	6.5%
	Generations and Gender Programme	2005	57.8%	27-85	1920-1978	1,766	1,536	13.0%	1,508	1.8%	1,445	4.2%
Spain	Occupational Prestige and Social Structure (Prestigio Ocupacional Y Estructura Social)	2013	51.4%	27-99	1914-1986	5,962	5,073	14.9%	5,032	0.8%	4,963	1.4%
Estonia	Generations and Gender Programme	2005	64.5%	27-81	1924-1978	7,855	7,035	10.4%	6,933	1.4%	6,922	0.2%
France	Training and Qualifying Survey Professional (Formation, Qualification Professionnelle)	1985	41.3%	27-84	1901-1958	39,233	30,724	21.7%	30,624	0.3%	30,243	1.2%
	Training and Qualifying Survey Professional (Formation, Qualification Professionnelle)	1993	50.8%	27-64	1929-1966	18,332	15,525	15.3%	15,244	1.8%	15,096	1.0%
	Training and Qualifying Survey Professional (Formation, Qualification Professionnelle)	2003	52.7%	27-65	1938-1976	39,285	33,514	14.7%	33,069	1.3%	32,859	0.6%
	Generations and Gender Programme	2005	55.7%	27-79	1926-1978	10,079	8,760	13.1%	8,740	0.2%	7,803	10.7%
United Kingdom	Oxford Social Mobility Inquiry	1972	0.0%	27-64	1902-1953	10,309	8,477	17.8%	8,424	0.6%	7,974	5.3%
	International Social Survey Program	1986	54.8%	35-85	1901-1951	1,416	961	32.1%	950	1.1%	-	100.0%
	UK National Survey of Sexual Attitudes and Life Style	1990	57.3%	27-59	1931-1963	4,548	3,474	23.6%	3,434	1.2%	-	100.0%

Country	Survey name	Survey year	% Female	Age range	Cohort range	No restriction	Restriction 1 (age 27 and over, birth cohorts 1901-1990)		Restriction 2 (non-missing on age, sex, educ., and sibsize)		Restriction 3 (non-missing on age, sex, educ., sibsize, and parent's educ.)	
						Sample size	Sample size	% Lost	Sample size	% Lost	Sample size	% Lost
	UK National Survey of Sexual Attitudes and Life Style	2000	58.5%	27-44	1955-1974	12,110	8,419	30.5%	8,417	0.0%	-	100.0%
	UK Household Longitudinal Study (Understanding Society)	2009-2011	58.1%	27-58	1951-1986	7,304	5,561	23.9%	5,057	9.1%	5,057	0.0%
	UK National Survey of Sexual Attitudes and Life Style	2010	59.7%	27-74	1935-1985	15,162	10,418	31.3%	10,408	0.1%	-	100.0%
Georgia	Generations and Gender Programme	2006	57.2%	27-80	1926-1979	10,000	8,359	16.4%	8,359	0.0%	7,852	6.1%
Hungary	International Social Survey Program	1986	55.0%	35-85	1901-1951	1,747	774	55.7%	755	2.5%	-	100.0%
	Social Stratification in Eastern Europe After 1989: General Population Survey	1993	54.0%	27-92	1901-1966	4,977	4,226	15.1%	4,071	3.7%	4,045	0.6%
	Generations and Gender Programme	2004	56.3%	27-79	1926-1978	13,540	11,828	12.6%	11,828	0.0%	11,673	1.3%
Italy	Generations and Gender Programme	2003	53.9%	27-64	1939-1976	9,570	8,518	11.0%	8,518	0.0%	8,318	2.3%
Japan	Japanese Social Stratification and Mobility	1995	53.7%	27-70	1925-1968	5,357	4,849	9.5%	2,978	38.6%	2,879	3.3%
	Japanese General Social Survey	2000	54.4%	27-89	1911-1973	2,893	2,636	8.9%	2,609	1.0%	2,182	16.4%
	Japan 2000 National Survey on Family and Economic Conditions	2000	52.6%	27-50	1950-1973	4,482	3,305	26.3%	2,463	25.5%	-	100.0%
	Japanese General Social Survey	2001	54.6%	27-89	1912-1974	2,790	2,566	8.0%	2,515	2.0%	2,085	17.1%
	Japanese General Social Survey	2002	53.8%	27-89	1913-1975	2,953	2,740	7.2%	2,717	0.8%	2,294	15.6%
	Japanese General Social Survey	2005	54.3%	27-89	1916-1978	2,023	1,873	7.4%	1,831	2.2%	1,407	23.2%
	Japanese Social Stratification and Mobility	2005	53.8%	27-70	1935-1978	5,742	5,317	7.4%	5,303	0.3%	4,405	16.9%
	Japanese General Social Survey	2006	53.4%	27-89	1917-1979	4,254	3,975	6.6%	3,914	1.5%	3,110	20.5%
	East Asian Social Survey	2006	54.9%	27-89	1917-1979	2,130	1,980	7.0%	1,951	1.5%	1,532	21.5%
	Japanese General Social Survey	2008	52.9%	20-89	1919-1988	4,220	4,220	0.0%	4,168	1.2%	3,475	16.6%
	National Family Research of Japan	2008	52.9%	28-73	1935-1980	5,203	5,203	0.0%	5,078	2.4%	4,304	15.2%
	Japan 2009 National Survey on Family and Economic Conditions	2009	52.8%	27-49	1960-1982	3,112	2,427	22.0%	2,064	15.0%	2,061	0.1%
	Japanese General Social Survey	2010	54.0%	27-89	1921-1983	5,003	4,675	6.6%	4,628	1.0%	3,789	18.1%
	Japanese General Social Survey	2012	54.0%	27-89	1923-1985	4,667	4,369	6.4%	4,323	1.1%	3,485	19.4%
S Korea	Inequality and Equity Survey	1990	17.5%	27-83	1907-1963	1,974	1,798	8.9%	1,798	0.0%	1,795	0.2%
	Korean General Social Survey	2004	54.3%	35-93	1911-1969	1,312	892	32.0%	890	0.2%	890	0.0%
	Korean General Social Survey	2006	56.4%	27-92	1914-1979	1,605	1,369	14.7%	1,334	2.6%	1,203	9.8%
	Education and Social Mobility Survey	2008-2011	49.6%	27-65	1943-1984	7,611	7,179	5.7%	7,178	0.0%	7,026	2.1%
Lithuania	Generations and Gender Programme	2006	51.1%	27-79	1926-1979	10,036	8,318	17.1%	8,274	0.5%	7,279	12.0%
The Netherlands	Family Survey of the Dutch Population (Family-enquete Nederlandse Bevolking)	1992	51.8%	27-78	1914-1965	1,801	1,649	8.4%	1,641	0.5%	1,638	0.2%
	Family Survey of the Dutch Population (Family-enquete Nederlandse Bevolking)	1998	49.9%	27-83	1915-1971	2,029	1,843	9.2%	1,842	0.1%	1,820	1.2%
	Family Survey of the Dutch Population (Family-enquete Nederlandse Bevolking)	2000	49.5%	27-84	1916-1973	1,561	1,445	7.4%	1,443	0.1%	1,434	0.6%
	Generations and Gender Programme	2003	57.9%	27-80	1923-1976	8,161	7,448	8.7%	7,435	0.2%	6,743	9.3%
	Family Survey of the Dutch Population (Family-enquete Nederlandse Bevolking)	2003	50.4%	27-79	1924-1976	2,174	1,980	8.9%	1,980	0.0%	1,945	1.8%
Norway	Generations and Gender Programme	2007	51.0%	27-81	1927-1981	14,880	12,999	12.6%	12,426	4.4%	12,205	1.8%
Poland	POLPAN (Polish Panel Survey)	1988	53.4%	27-68	1920-1961	5,817	5,281	9.2%	1,720	67.4%	1,689	1.8%

Country	Survey name	Survey year	% Female	Age range	Cohort range	No restriction	Restriction 1 (age 27 and over, birth cohorts 1901-1990)		Restriction 2 (non-missing on age, sex, educ., and sibsize)		Restriction 3 (non-missing on age, sex, educ., sibsize, and parent's educ.)	
						Sample size	Sample size	% Lost	Sample size	% Lost	Sample size	% Lost
	Social Stratification in Eastern Europe After 1989: General Population Survey	1993	52.9%	27-77	1916-1966	3,520	2,932	16.7%	2,846	2.9%	2,794	1.8%
	Polish General Social Survey	1997	56.5%	27-92	1905-1970	2,401	2,057	14.3%	1,018	50.5%	984	3.3%
	Polish General Social Survey	1999	56.5%	27-94	1905-1972	2,282	1,942	14.9%	1,936	0.3%	1,892	2.3%
	Polish General Social Survey	2002	58.2%	27-94	1908-1975	2,473	2,181	11.8%	2,180	0.0%	2,166	0.6%
	Generations and Gender Programme	2010	58.5%	27-83	1927-1984	19,987	17,533	12.3%	16,693	4.8%	15,661	6.2%
Romania	Generations and Gender Programme	2005	50.6%	27-80	1925-1978	11,986	10,878	9.2%	10,878	0.0%	10,677	1.8%
Russia	Russian General Social Survey	1992	56.7%	27-89	1903-1965	2,149	1,694	21.2%	1,646	2.8%	-	100.0%
	Social Stratification in Eastern Europe After 1989: General Population Survey	1993	60.7%	27-89	1904-1966	5,002	4,191	16.2%	3,698	11.8%	3,633	1.8%
	Generations and Gender Programme	2004	63.7%	27-81	1923-1977	11,261	9,614	14.6%	8,898	7.4%	7,668	13.8%
Sweden	Generations and Gender Programme	2012	51.8%	27-80	1933-1986	9,688	8,188	15.5%	8,153	0.4%	7,580	7.0%
Taiwan	Taiwan Social Change Survey	1996	46.1%	27-76	1919-1968	1,924	1,748	9.1%	1,739	0.5%	1,684	3.2%
	Taiwan Social Change Survey	1997	53.2%	27-64	1933-1970	1,717	1,465	14.7%	1,461	0.3%	1,426	2.4%
	Panel Study of Chinese Family Dynamics	1999	54.8%	36-46	1953-1963	999	999	0.0%	995	0.4%	973	2.2%
	Taiwan Social Change Survey	2001	50.3%	27-93	1902-1968	1,979	1,753	11.4%	1,752	0.1%	1,701	2.9%
	Taiwan Social Change Survey	2004	49.0%	27-98	1906-1977	1,781	1,462	17.9%	1,462	0.0%	1,418	3.0%
	Taiwan Social Change Survey	2006	51.2%	27-92	1903-1968	2,102	1,749	16.8%	1,748	0.1%	1,714	1.9%
	Taiwan Social Change Survey	2012	50.9%	27-101	1911-1985	2,134	1,825	14.5%	1,821	0.2%	1,755	3.6%
	Taiwan Social Change Survey	2014	51.0%	27-97	1917-1987	1,875	1,599	14.7%	1,506	5.8%	1,465	2.7%
	Taiwan Social Change Survey	2015	48.9%	27-94	1921-1988	2,034	1,731	14.9%	1,728	0.2%	1,664	3.7%
United States	Growth of American Families	1955	100.0%	27-54	1901-1928	2,713	2,218	18.2%	2,218	0.0%	-	100.0%
	General Social Survey	1972-2014	56.1%	27-89	1901-1987	59,599	49,937	16.2%	48,344	3.2%	43,125	10.8%
Total						695,693	577,378	17.0%	545,110*	5.6%	462,827*	15.1%

Notes: * These numbers differ from what Table B1 reports (536,124 and 453,173) because some observations that are dropped due to small cohort×country cell size (<500) are still included in this table.

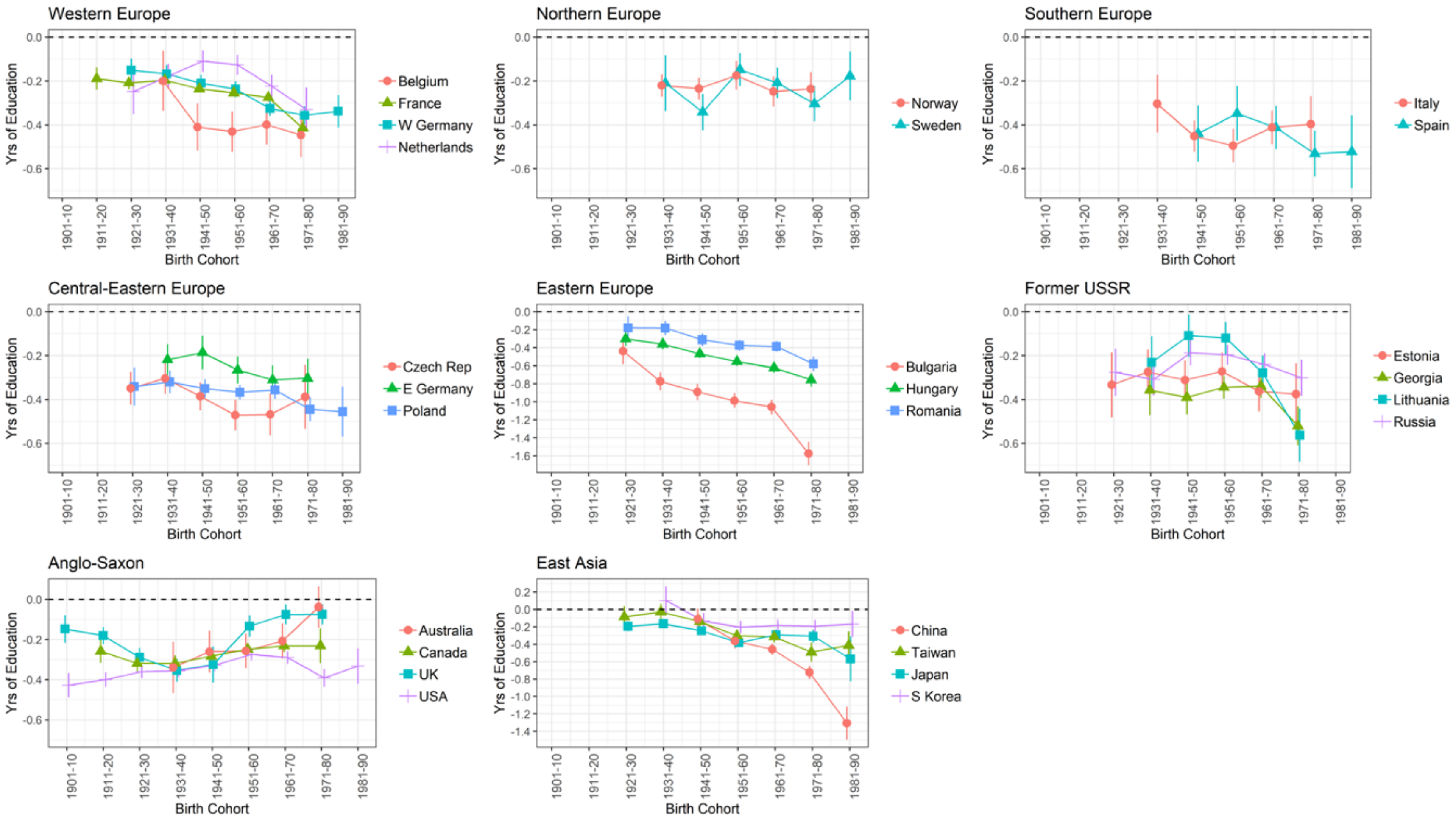

Fig.B1 Cohort trends in disadvantage in educational attainment from having an additional sibling (nested samples): whiskers show 95 confident intervals of the coefficient. Note that the scale of y-axis is different for Eastern Europe and East Asia due to Bulgaria and China.

C: Results from Supplementary Analyses

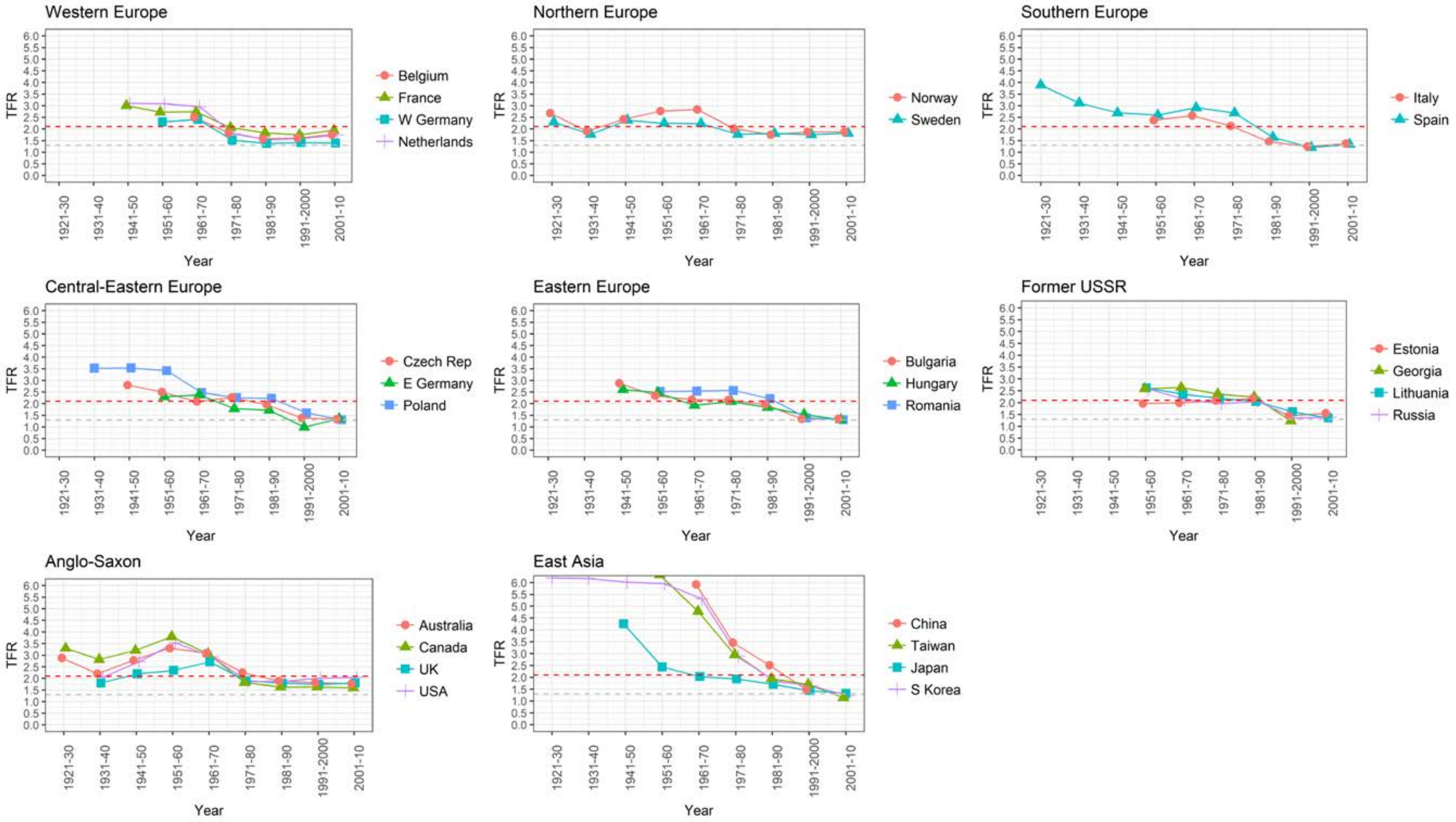

Fig.C1 Trends of total fertility rate (TFR): red dashed line indicates the replacement level of fertility (TFR 2.1). Grey dashed line indicates the lowest low level of fertility (TFR 1.3). Sources: Human Fertility Database (https://www.humanfertility.org) and Human Fertility Collection (https://www.fertilitydata.org/).

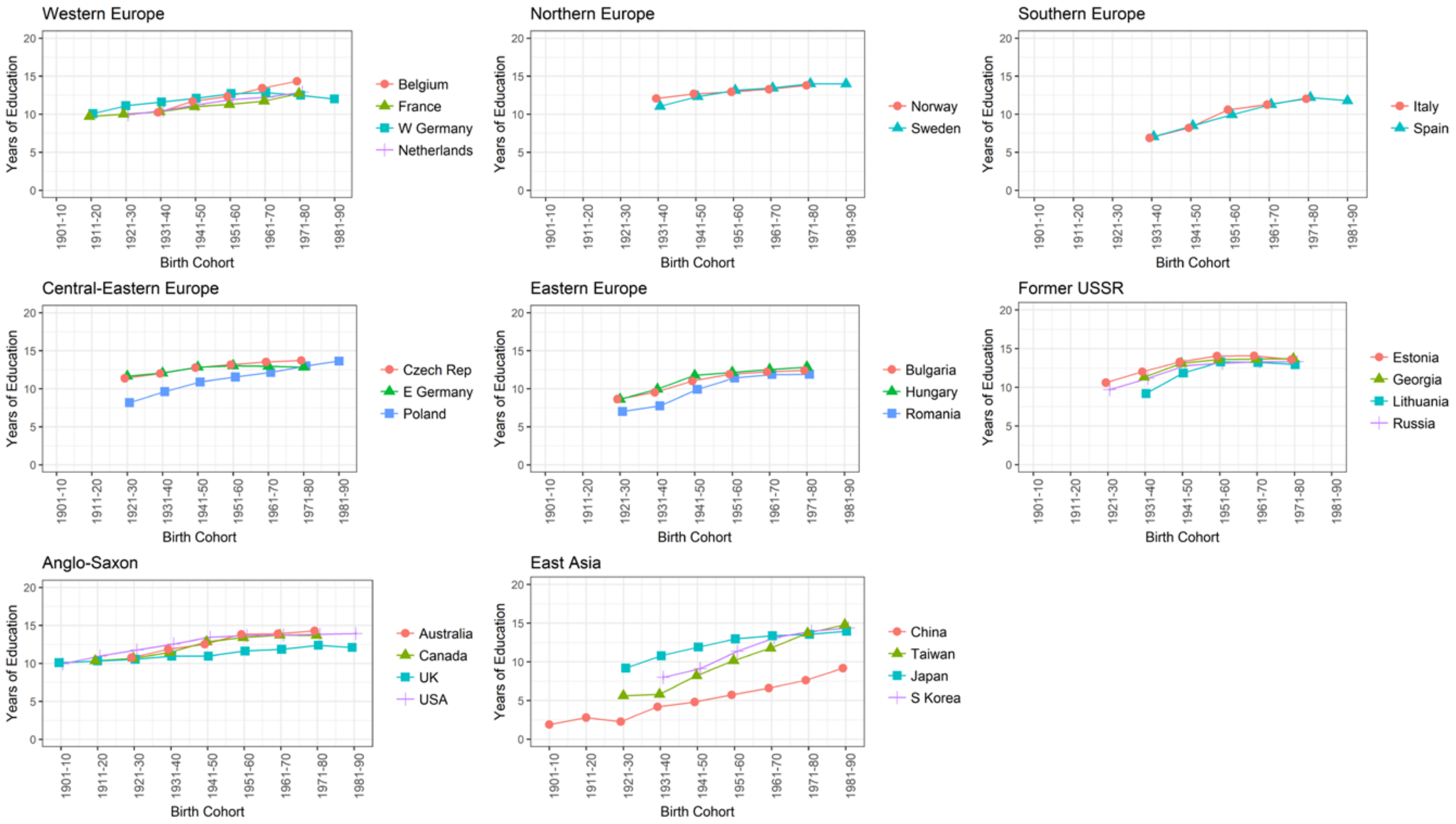

Fig. C2 Cohort trends of educational attainment: see https://dataverse.harvard.edu/dataverse/isead for the comparable trends based on other country-level statistics, which shows consistency with this figure with minor differences.

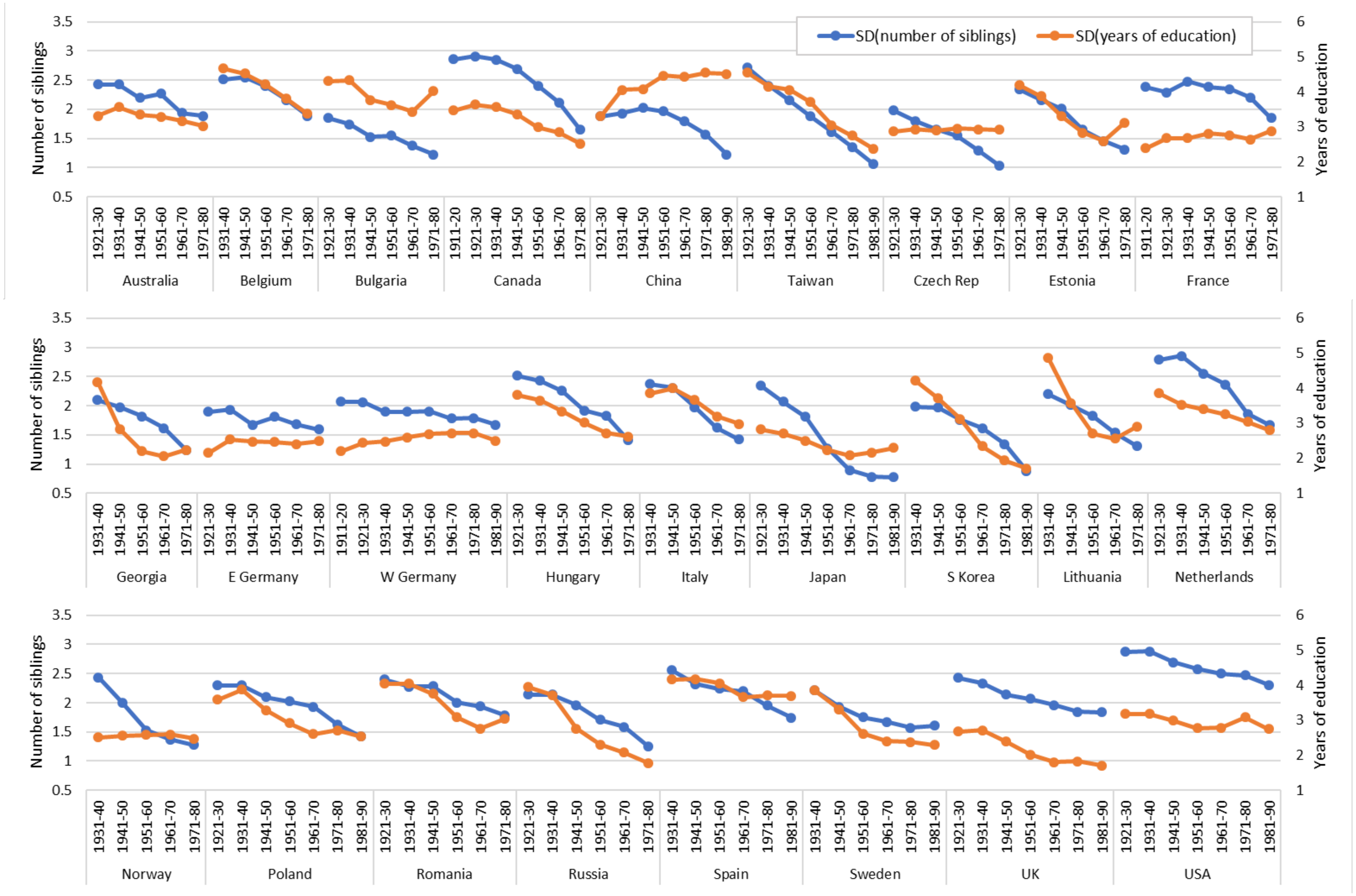

Fig.C3 Cohort trends in the dispersions of sibship size and educational attainment

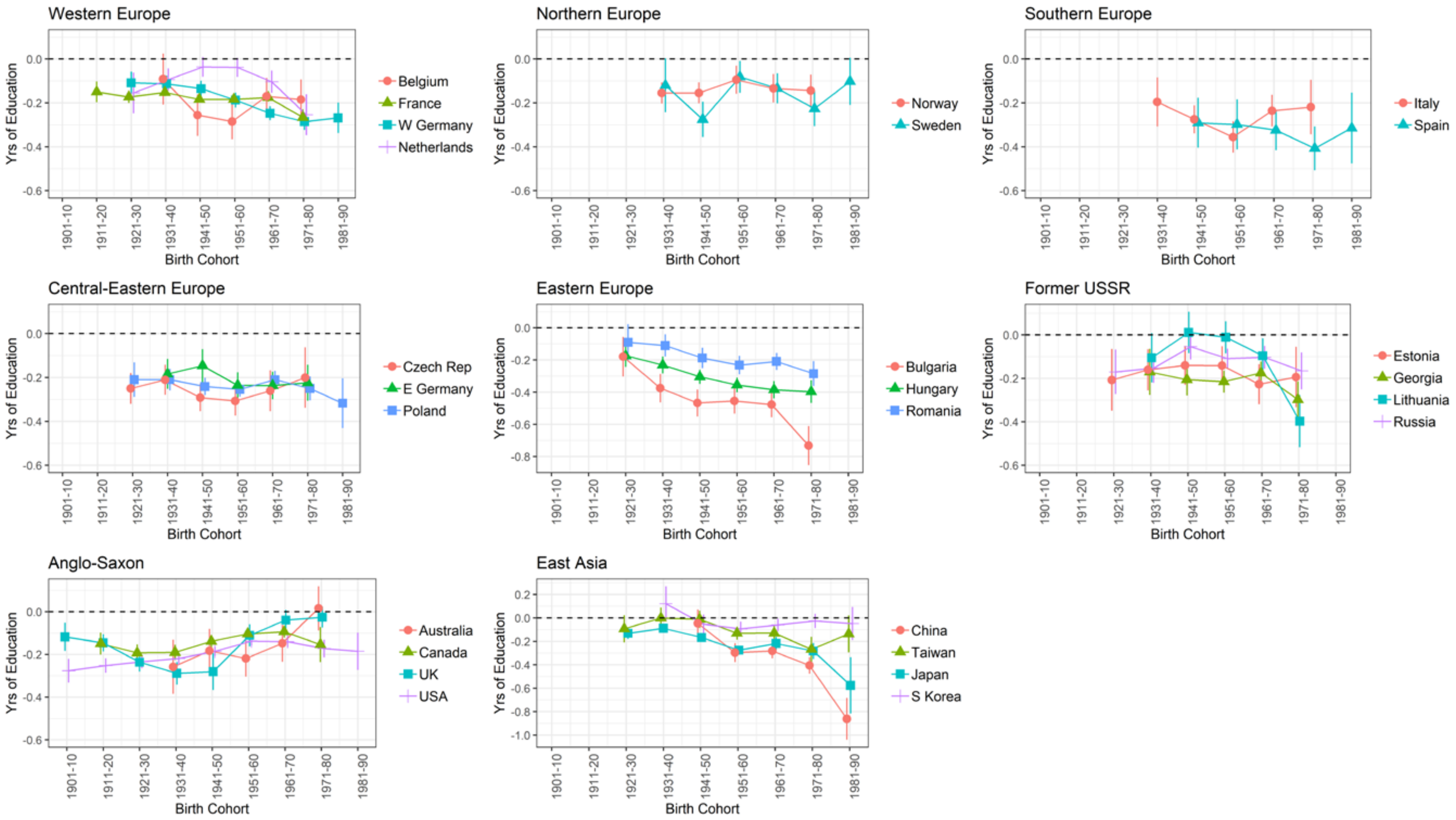

Fig. C4 Cohort trends in disadvantage in educational attainment from having an additional sibling net of parental education: whiskers show 95 confident intervals of the coefficient. Note that the scale of y-axis is different for Eastern Europe and East Asia due to Bulgaria and China.

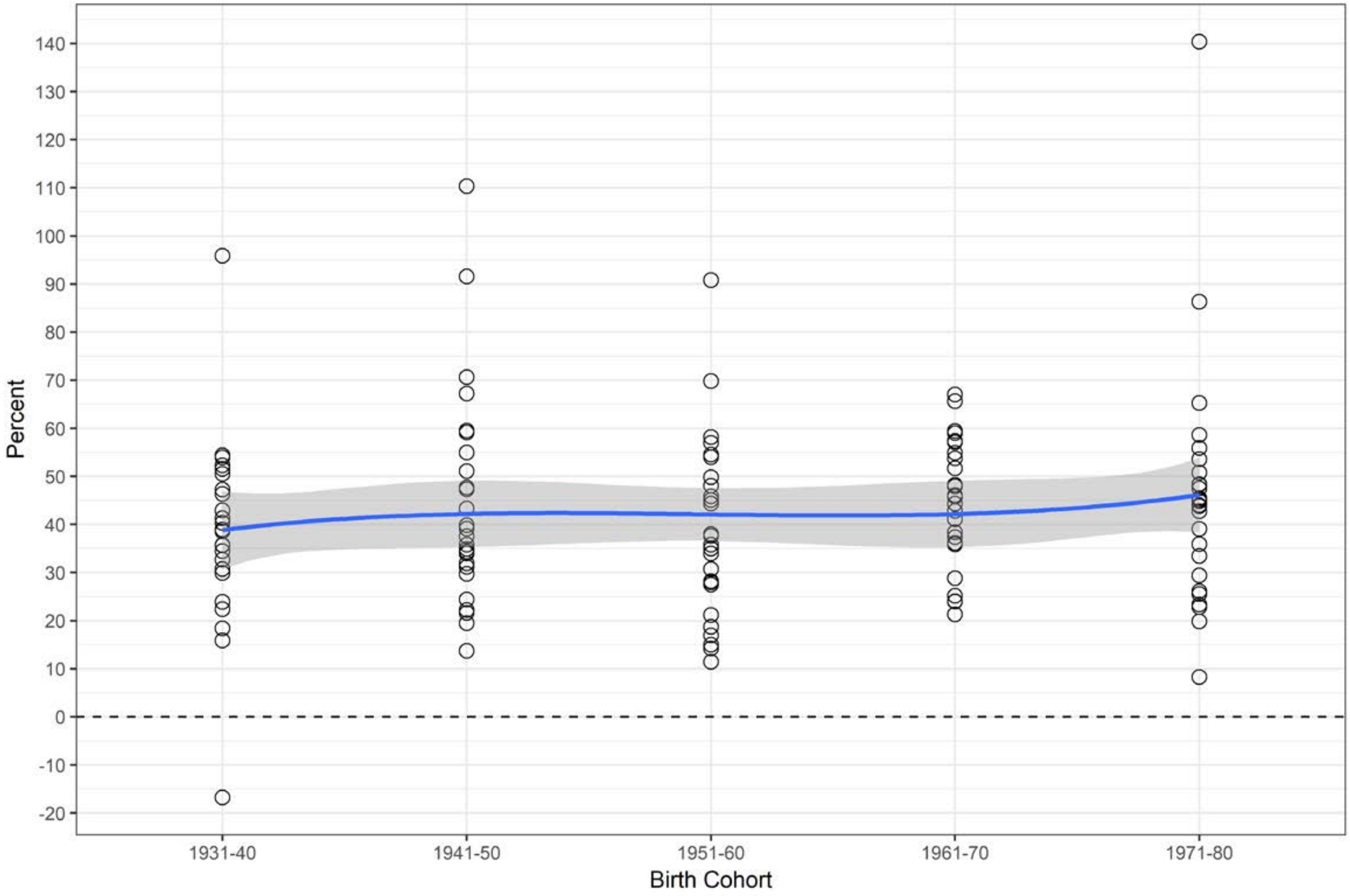

Fig.C5 Percentage reduction in educational attainment associated with an additional sibling, conditioning on parental education

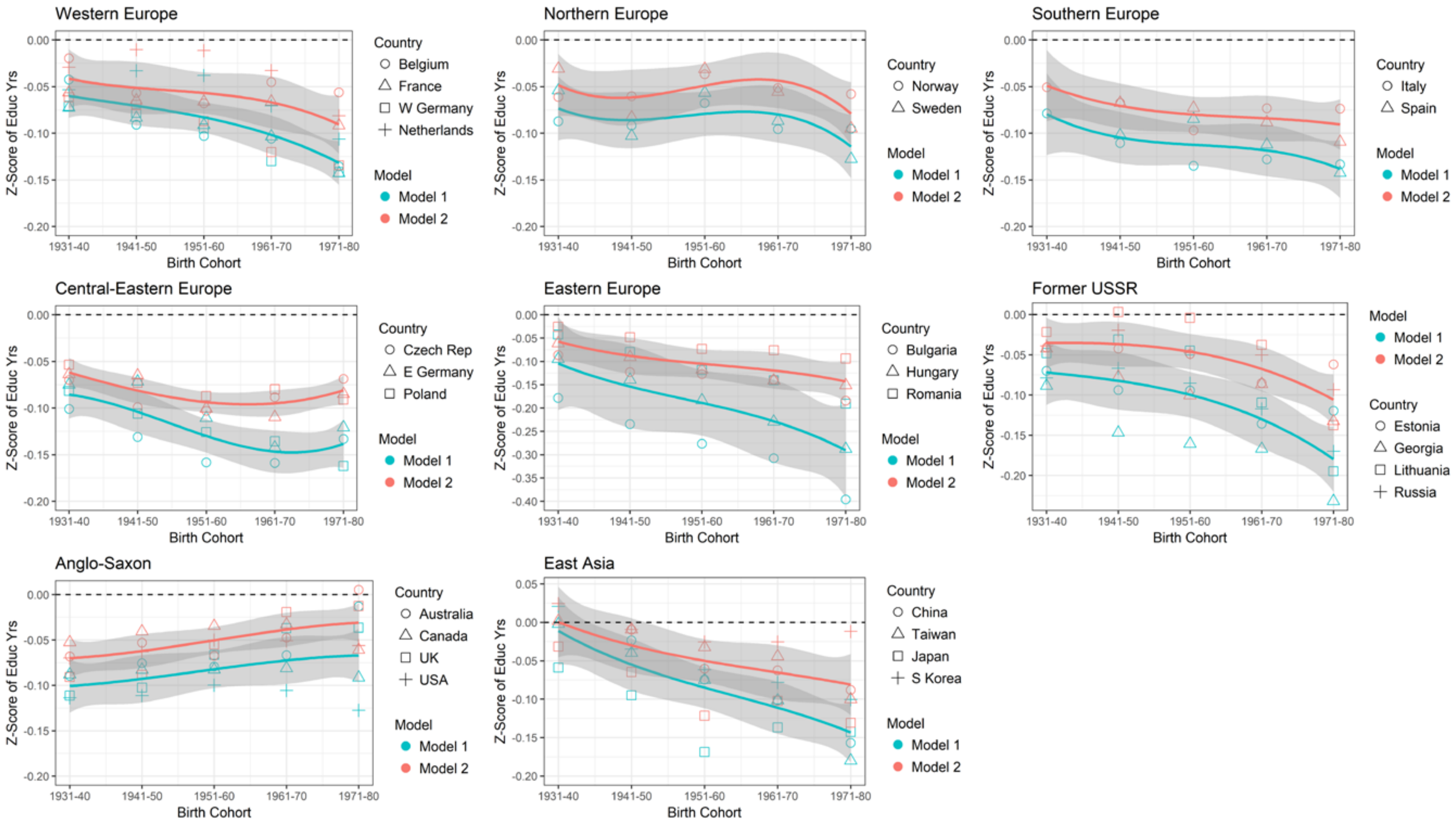

Fig.C6 Relative education by an additional sibling, with and without conditioning on parental education: grey areas show 95 confident intervals of fitted curves. Note that the scale of y-axis is different for Eastern Europe due to Bulgaria.

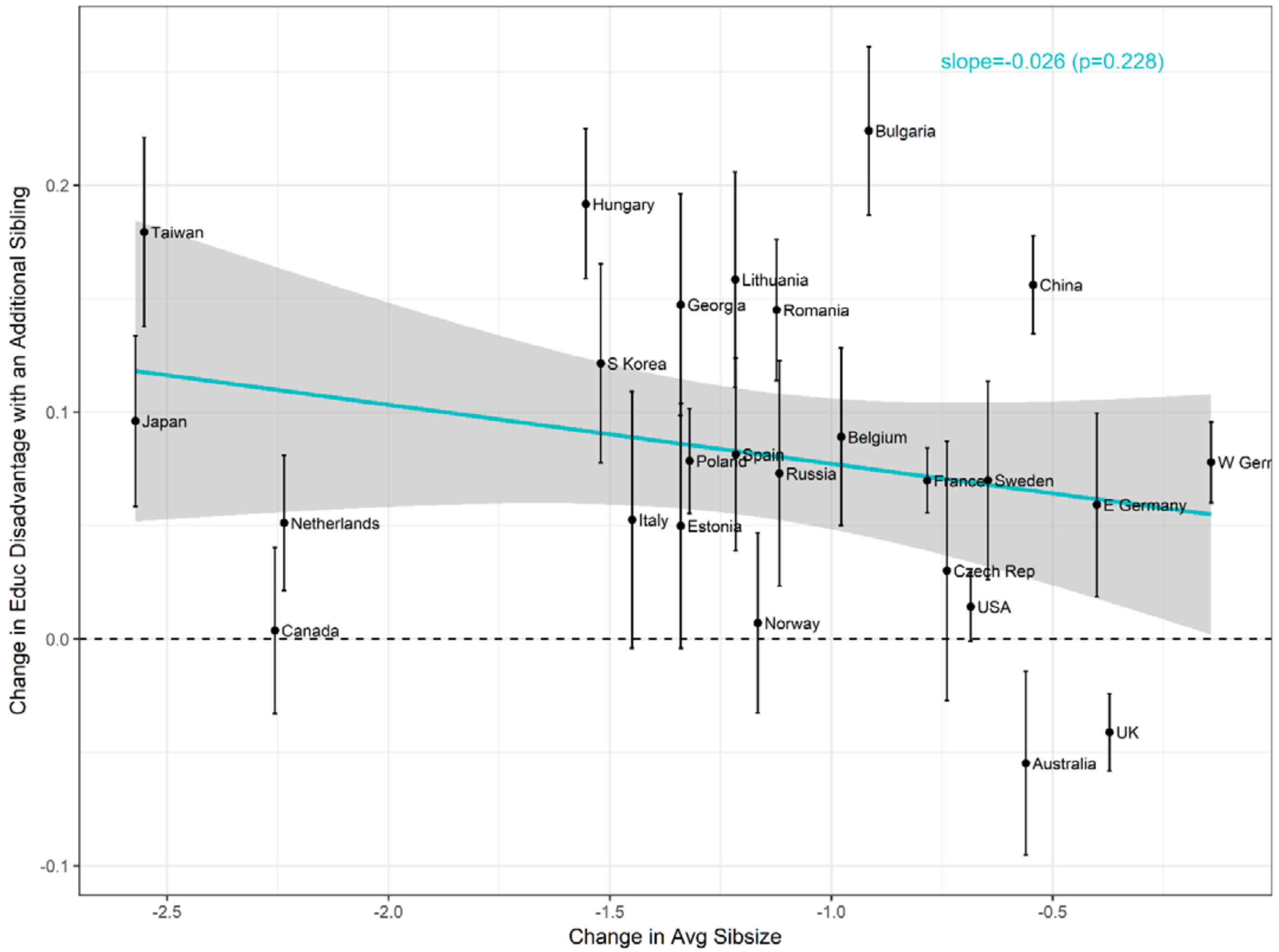

Fig.C7 Cross-national association between change in average sibship size and change in disadvantage in relative education associated with an additional sibling

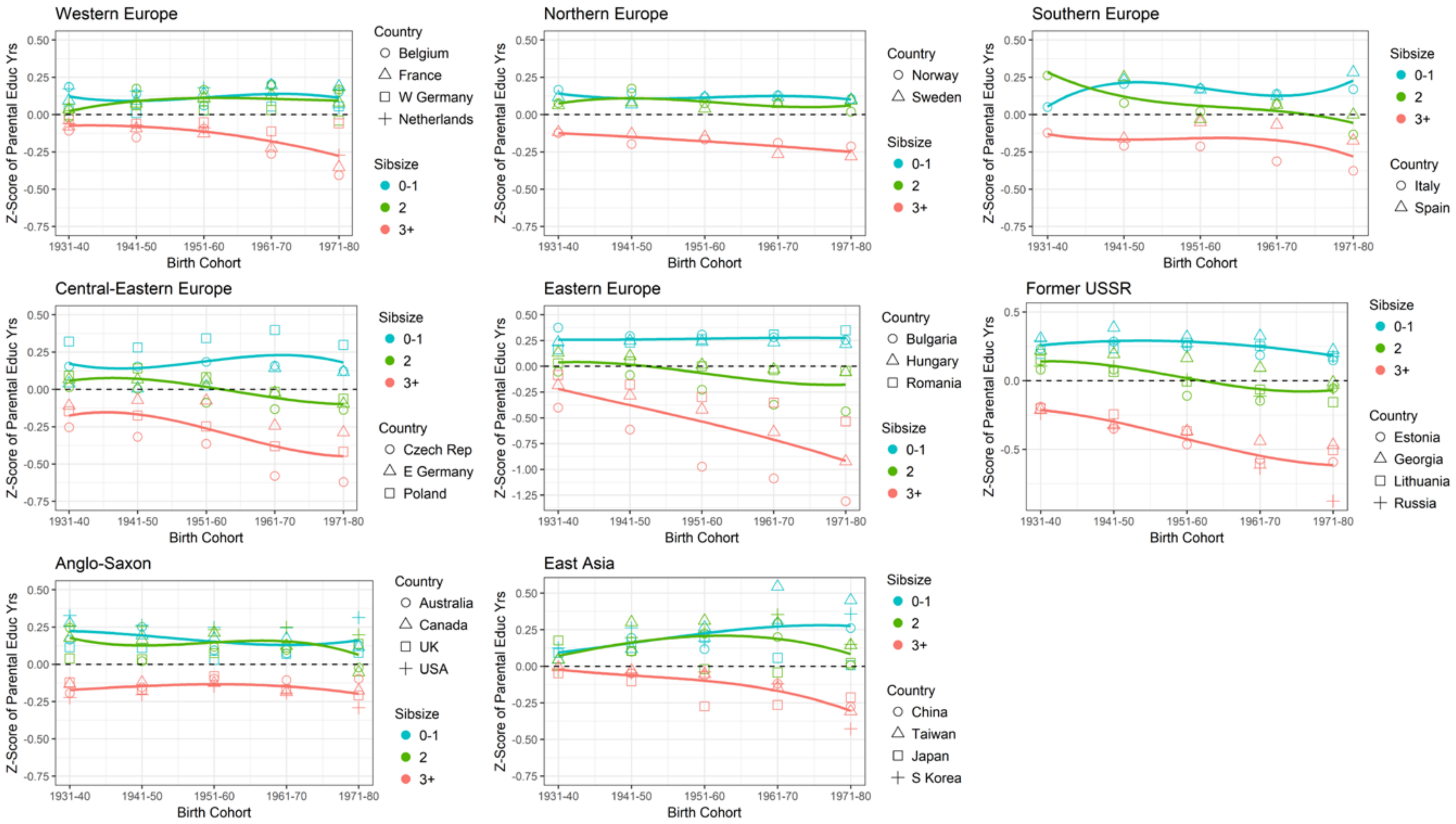

Fig.C8 Average parental relative education completed, by sibship size: note that the scale of y-axis is different for Eastern Europe due to Bulgaria.

D: Results from Robustness Analyses

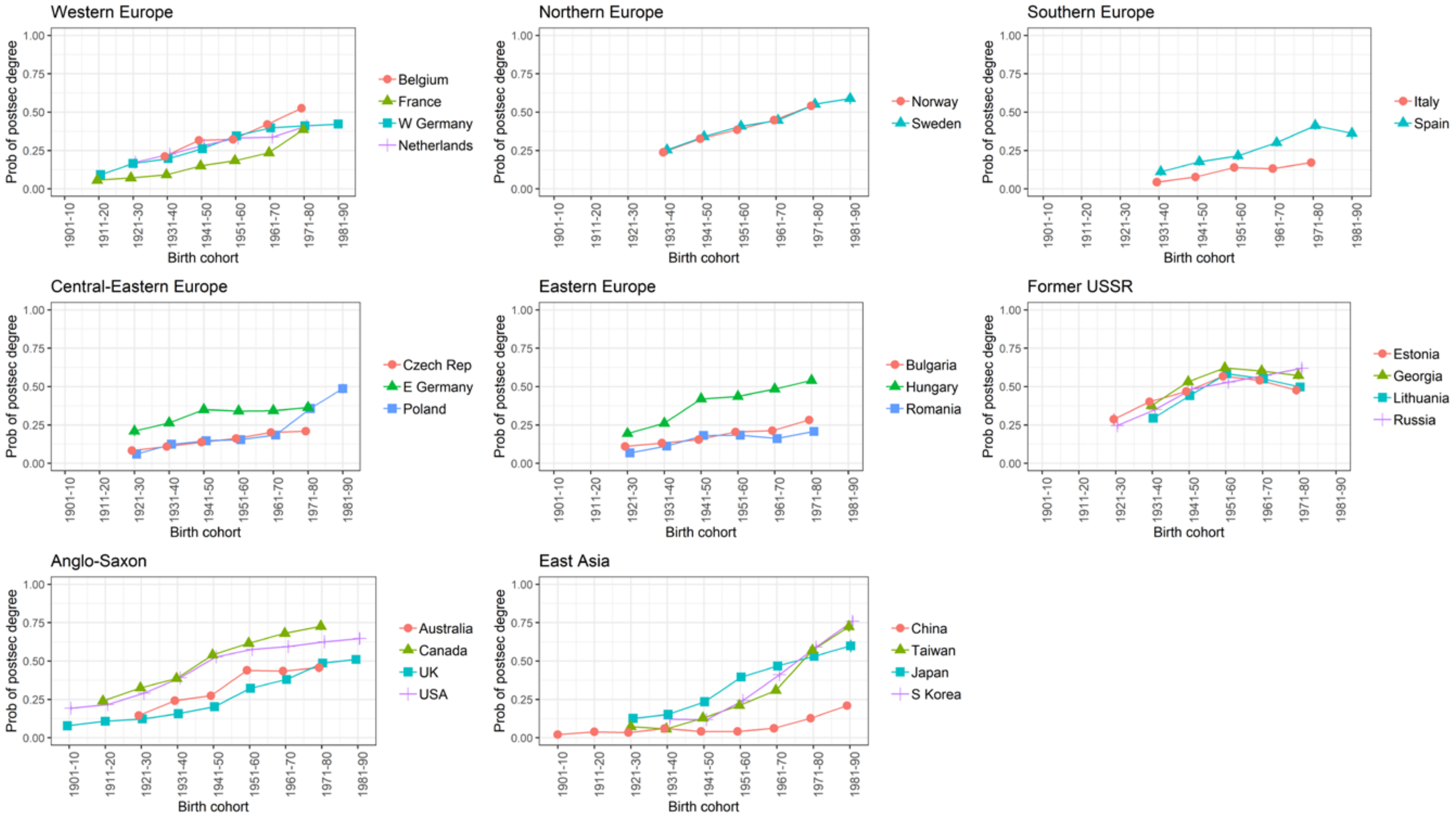

Fig. D1 Cohort trends of the probability of attaining postsecondary degree: see https://dataverse.harvard.edu/dataverse/isead for the comparable trends based on other country-level statistics, which shows consistency with this figure with a couple of notable exceptions (e.g., Hungary and Russia)

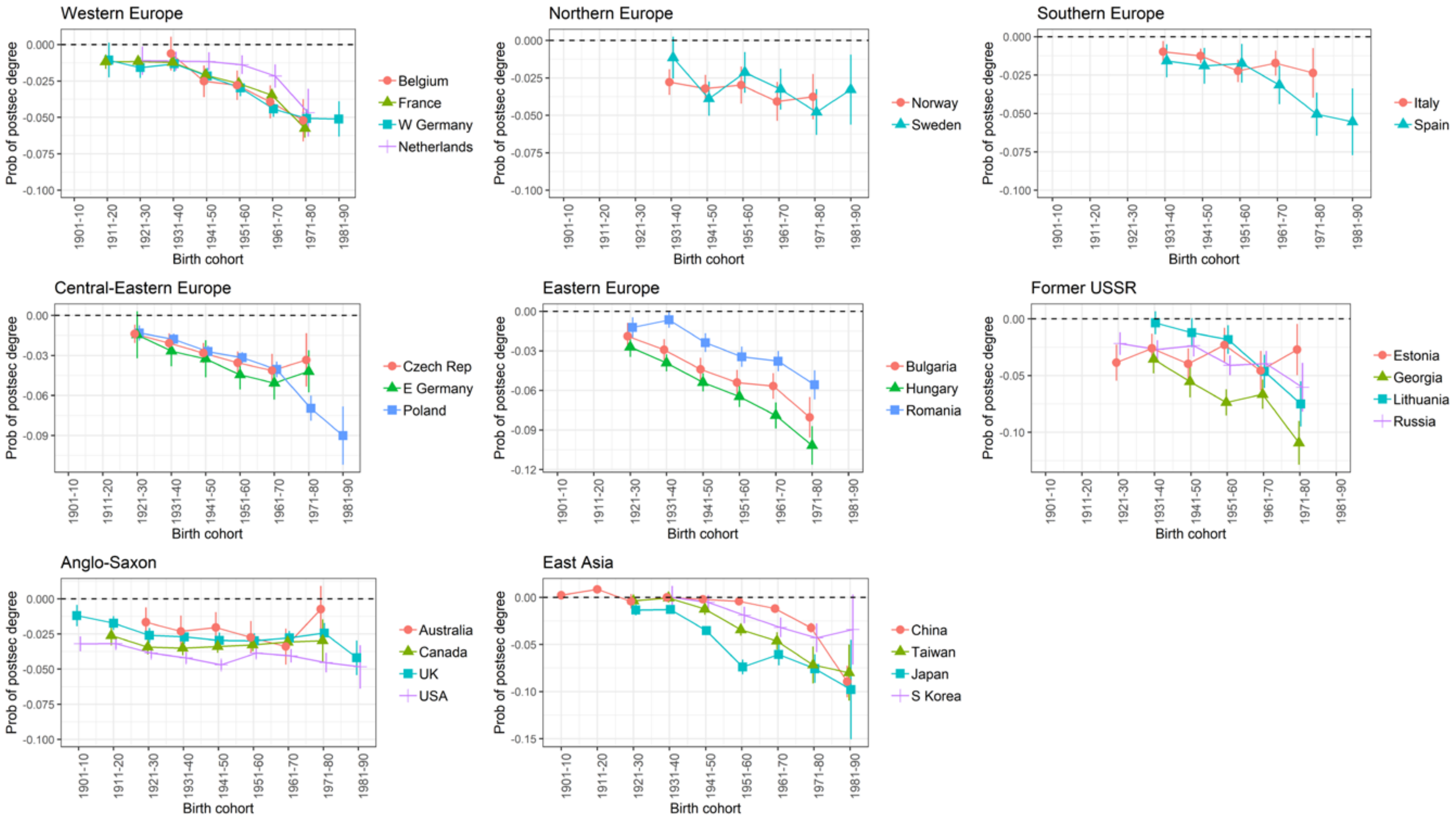

Fig.D2 Cohort trends in disadvantage in attaining postsecondary degree from having an additional sibling: whiskers show 95 confident intervals of the coefficient.

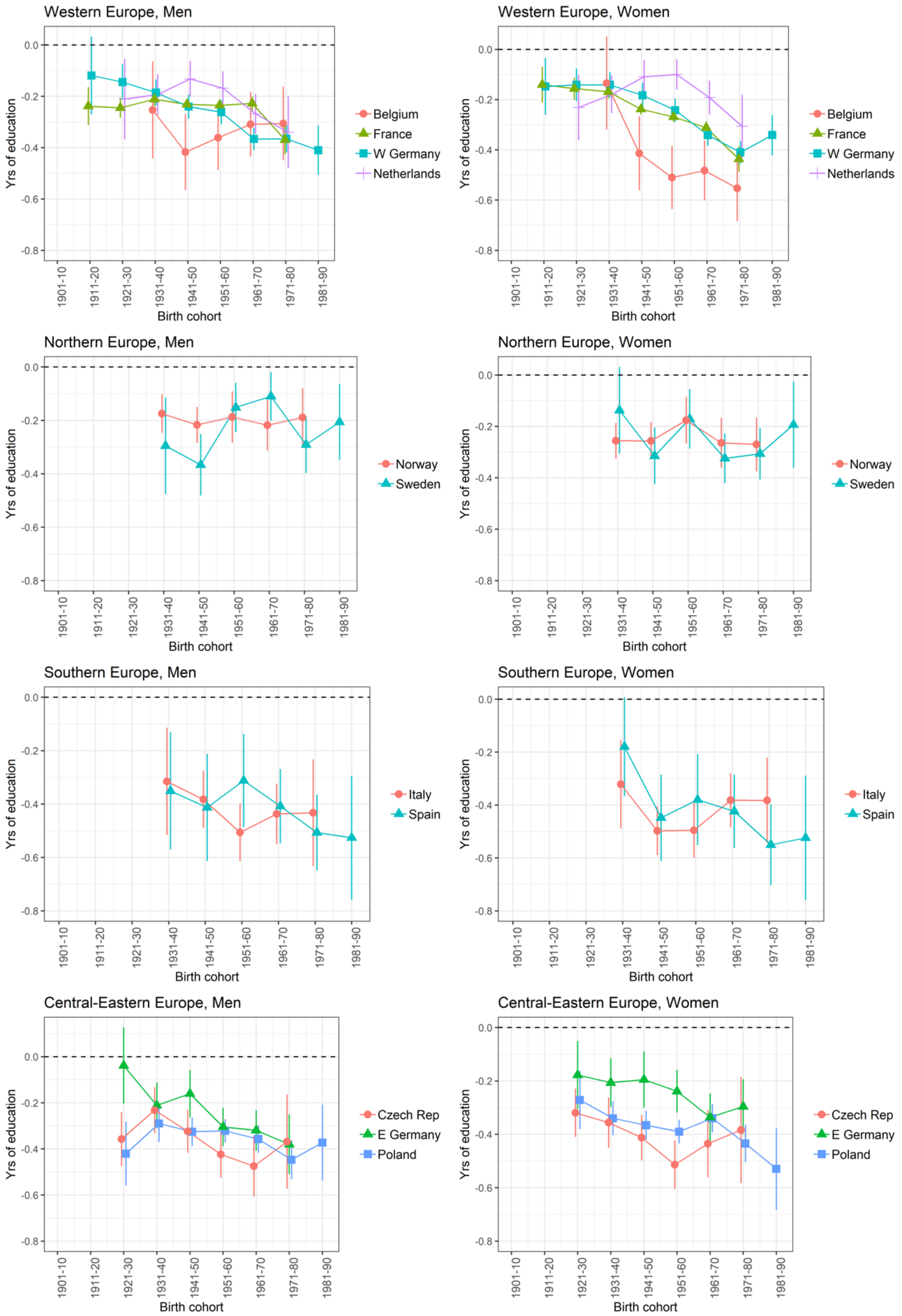

Fig.D3 Cohort trends in sibship size disadvantage in educational attainment, by gender

Fig.D3 (continued)

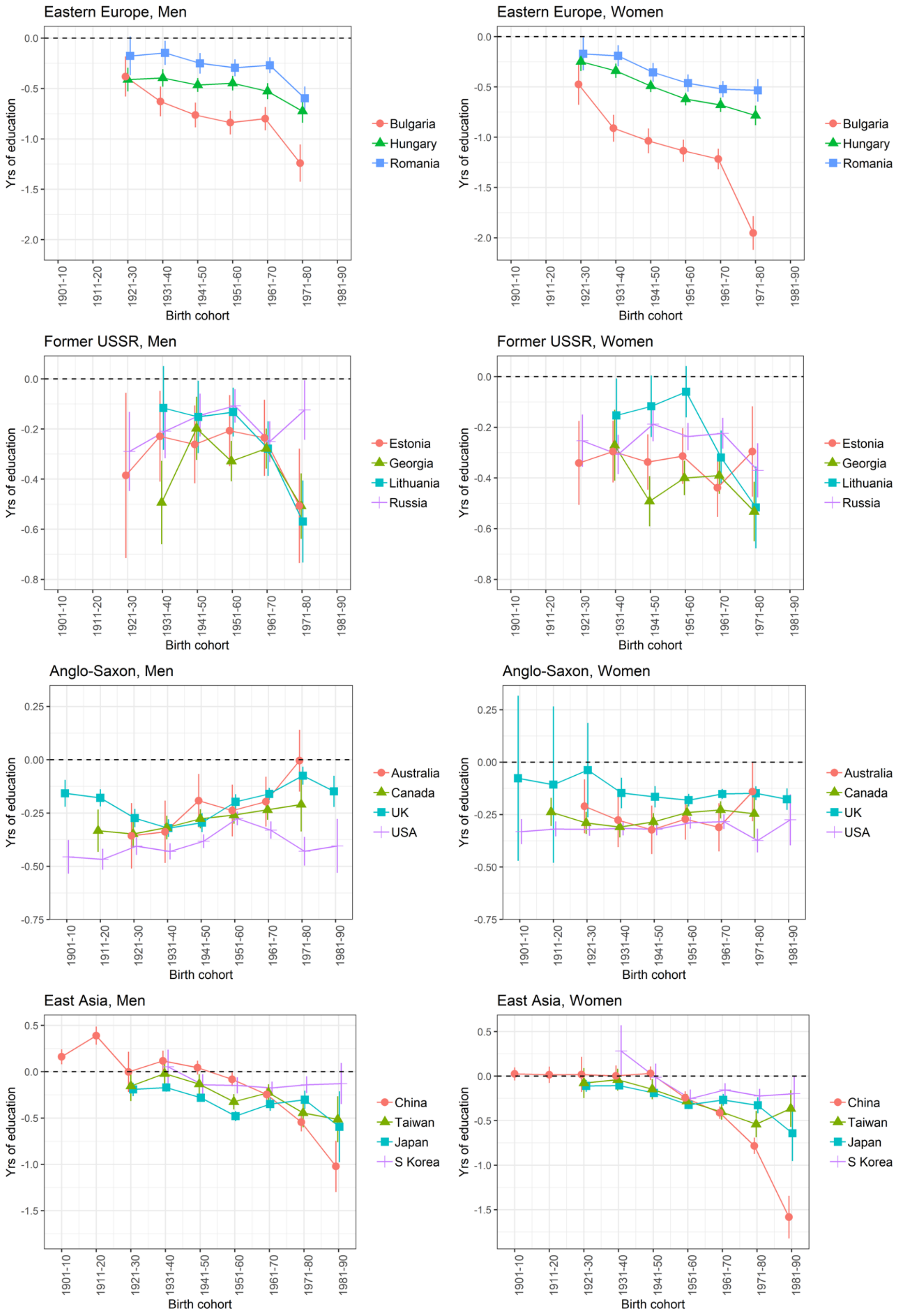

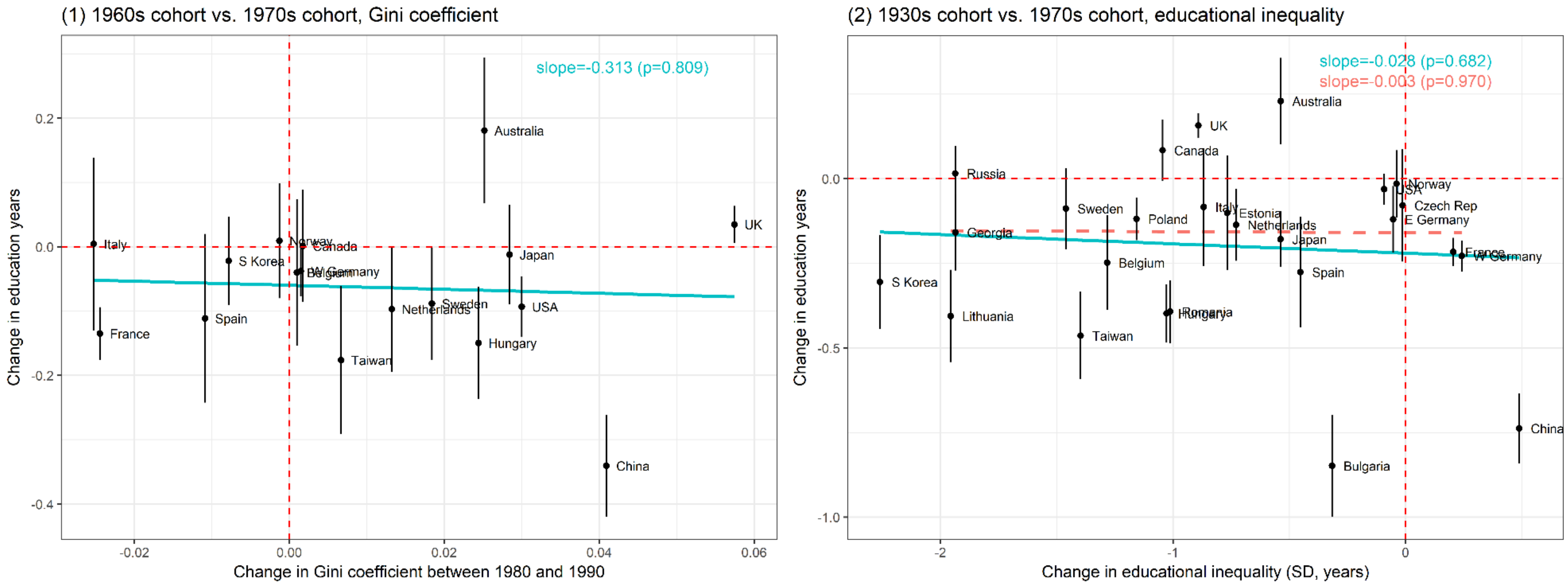

Fig.D4 Changes in economic and educational inequalities and cohort changes in sibship size disadvantage from having an additional sibling: the red dashed line shows the fitted line when East Asian countries are excluded. Gini coefficients are measured by the three-year averages around the beginning year (1980) and the end year (1990) of the comparison. These years, 1980 and 1990, capture the teenage years of the 1960s cohort and the 1970s cohort. This short interval of the cohort comparison is due to data availability of the Gini coefficient across sampled countries. The source of Gini coefficient data is Standardized World Income Inequality Database (Gini coefficient of disposable household income) (Solt 2016, https://fsolt.org/swiid/).
